# Investigating the Dynamics of a Soft Crystalline Covalent Organic Framework during Benzene and Cyclohexane Adsorption by in situ Powder X‐ray Diffraction

**DOI:** 10.1002/smsc.202400277

**Published:** 2024-10-12

**Authors:** Anna Mauri, Rebecca Vismara, Marco Moroni, Esther Roldán‐Molina, Jorge A. R. Navarro, Simona Galli

**Affiliations:** ^1^ Dipartimento di Scienza e Alta Tecnologia Università degli Studi dell’Insubria Via Valleggio 9 22100 Como Italy; ^2^ Departamento de Química Inorgánica Universidad de Granada Avenida Fuentenueva S/N 18071 Granada Spain; ^3^ Dipartimento di Chimica Università degli Studi di Pavia Via Taramelli 12 27100 Pavia Italy; ^4^ Consorzio Interuniversitario Nazionale per la Scienza e la Tecnologia dei Materiali Via Giusti 9 50121 Firenze Italy

**Keywords:** benzene/cyclohexane separation, covalent organic frameworks, in situ powder X‐ray diffraction, soft porous crystals

## Abstract

Due to their similar boiling points, separation of benzene and cyclohexane mixtures is among the current challenging processes faced by the petrochemical industry. As recently assessed, the soft imine‐based covalent organic framework [(TAM)(BDA)_2_] (COF‐300; TAM = tetrakis(4‐aminophenyl)methane, BDA = terephthaldehyde) possesses higher affinity for benzene than cyclohexane in both static conditions at 298 K and dynamic conditions in the range of 298–348 K. As shown in this contribution, in situ powder X‐ray diffraction while dosing benzene and cyclohexane vapors in the range of 0.01–4.74 bar on the narrow‐pore form of COF‐300 confirmed the coherent switchability of its framework, unveiling the progressive formation of different intermediate‐ and large‐pore forms. In addition, a basket of otherwise inaccessible key crystallochemical details—“on/off” structural‐feature changes cooperating to adsorption, primary adsorption sites, and host–guest and guest–guest interactions—was successfully retrieved. Overall, these findings allowed to shed light on the framework dynamics underneath the previously observed selectivity toward benzene over cyclohexane, completing this case of study and providing relevant information for the design of new‐generation adsorbents for this applicative context.

## Graphical abstract

*In situ* powder X‐ray diffraction confirms the switchability of the covalent organic framework (COF)‐300 during benzene and cyclohexane adsorption. Starting from the narrow‐pore form, intermediate‐ and large‐pore forms, as well as structural changes and host ‐guest interactions favoring adsorption, are identified. Disclosing the framework dynamics underneath COF‐300 selectivity toward benzene provides key information for new‐generation adsorbents in this applicative context.

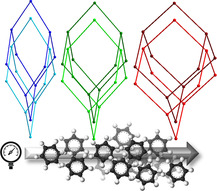

## 1 Introduction

Single‐crystal and powder X‐ray diffraction^[^ ^1^ ^]^ as well as electron diffraction^[^ ^2^ ^]^ are the most common non‐destructive characterization methods to study long‐ and short‐range structural features of nanoporous materials such as metal‐organic frameworks (MOFs)^[^ ^3^, ^4^, ^5^ ^]^ and covalent organic frameworks (COFs).^[^ ^6^, ^7^, ^8^ ^]^ With bulk materials, to go beyond crystal structure determination and microstructure assessment, in situ powder X‐ray diffraction under non‐ambient conditions (NA‐PXRD) offers a portfolio of cutting‐edge tools to unveil key crystallochemical details contributing to rationalize the functional behavior of the current case of study, and to optimize, for the investigated applicative context, the class of materials to which the case belongs. In the past two decades, NA‐PXRD methods have known an incessant development in the study of nanoporous materials.^[^ ^1^ ^]^ As shown by different research groups^[^ ^1^, ^9^, ^10^, ^11^ ^]^ and by us,^[^ ^12^, ^13^, ^14^ ^]^ coupling NA‐PXRD to synchrotron radiation,^[^ ^15^ ^]^ granting higher brilliance and signal‐to‐noise ratio as well as faster measurement times than in‐house instrumentation, can be extremely beneficial in MOFs thorough characterization.

Numerous examples^[^ ^16^ ^]^ exist, in the realm of MOFs, of soft porous crystals,^[^ ^17^ ^]^ combining periodic homogeneity with flexibility, while still few are the study cases^[^ ^18^, ^19^, ^20^, ^21^ ^]^ involving COFs. This occurrence is reasonably due to the higher energy of covalent bonds, which makes COFs polymeric frameworks stiffer, in response to external stimuli, than MOFs, characterized by coordinat metal‐to‐ligand coordination bonds. COFs feature 2D^[^ ^22^ ^]^ or 3D^[^ ^23^ ^]^ open frameworks built through the condensation of organic monomers via covalent bonds. Imine‐based COFs^[^ ^24^ ^]^ show a number of advantages with respect to other COF families: they can be isolated in a variety of experimental conditions, including room temperature, and typically possess higher chemical stability.

**Scheme 1:**
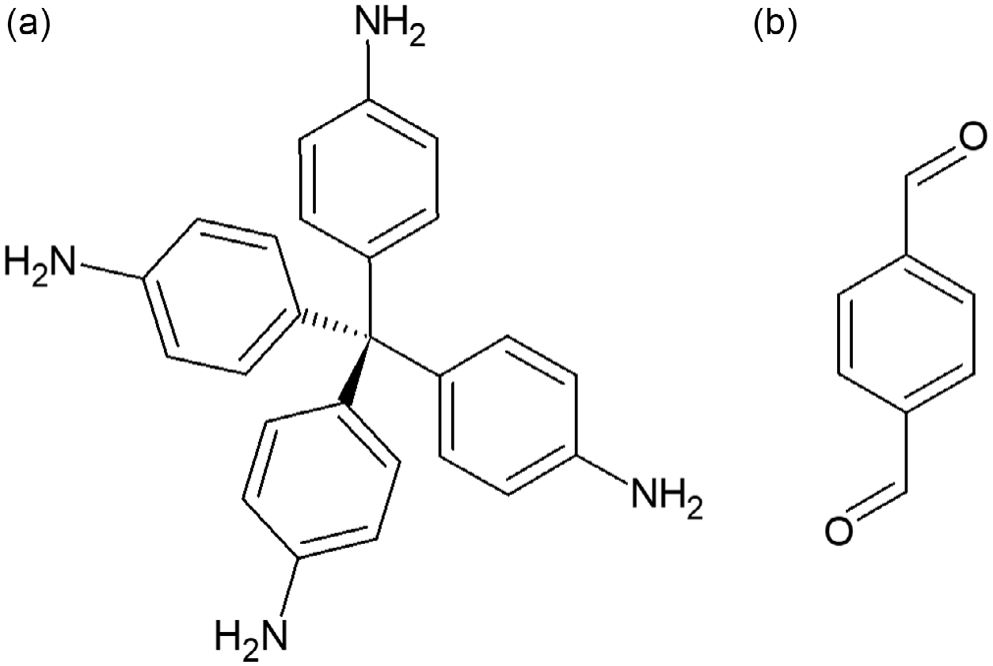
Molecular structure of a) tetrakis(4‐aminophenyl)methane (TAM) and b) terephthaldehyde (BDA).

In this context, with the present work, we demonstrate that synchrotron‐radiation NA‐PXRD in situ dosing benzene and cyclohexane vapors can be successfully employed to identify the crystallochemical features underneath the higher affinity for benzene of the 3D imine‐based COF [(TAM)(BDA)_2_] (COF‐300; TAM = tetrakis(4‐aminophenyl)methane, BDA =terephthaldehyde, **Scheme** **1**). First reported in 2009,^[^ ^25^ ^]^ COF‐300 conjugates periodic homogeneity to crystal structure stability and flexibility. In the past, a narrow‐pore form (NP, also named hydrated,^[^ ^26^ ^]^ collapsed,^[^ ^27^ ^]^ or COF‐300‐H_2_O^[^ ^28^ ^]^) and a large‐pore form (LP^[^ ^26^ ^]^ or COF‐300‐THF^[^ ^28^ ^]^) have been identified, as a function of the adsorbate, and characterized. Recently, we exploited COF‐300 to investigate the role of COFs softness in the selective adsorption of benzene over cyclohexane in both static and dynamic conditions.^[^ ^29^ ^]^ The two chemicals share very similar boiling points (353.2 and 353.9 K at ambient pressure for benzene and cyclohexane, respectively), making their separation by means of fractional distillation unfeasible, irrespective of the benzene:cyclohexane ratio of the mixture.^[^ ^30^ ^]^ At present, azeotropic distillation and extractive distillation are employed at the industrial level to separate the two chemicals. Nonetheless, both processes are complex and energy consuming. The currently investigated separation techniques based on selective adsorption could represent a key improvement, considerably reducing energy consumption.

As we reported previously,^[^ ^29^ ^]^ at 298 K and up to 1 bar, the NP form of COF‐300 adsorbs more benzene than cyclohexane (251 vs 175 cm^3^ g^−1^ at standard pressure and temperature (STP), respectively). The two isotherms are characterized by both hysteresis loops and steps, as expected with a soft material. In addition, flowing a 50:50 v v^−1^ mixture of the two chemicals in the range of 298–348 K, the NP form exhibits a higher retention time for benzene. Particle size appeared not to influence this specific performance.

To rationalize these observations, we recurred to high‐resolution NA‐PXRD (HR‐NA‐PXRD) at the beamline ID22 of the European Synchrotron Radiation Facility (ESRF). As reported in the following, in situ dosing benzene or cyclohexane vapors on the NP form of COF‐300, not only we confirmed the higher affinity of this adsorbent for the former chemical but, also and more importantly, we gained a basket of crystallochemical features unveiling 1) a dynamic, coherently switchable system of higher complexity with respect to what could be inferred based on the NP and LP forms alone, undergoing both gradual and “on/off” guest‐triggered modifications of the framework; 2) the primary adsorption sites, disclosing unexpected host–guest interactions in pores whose walls are decorated with aromatic rings.

## 2 Results and Discussion

### 2.1 Brief Reviewing of COF‐300 Synthesis and Structural Aspects

As reported in Section 4.2, for the present study, we isolated COF‐300 according to the synthetic path we recently optimized^[^ ^29^ ^]^ to recover the NP form as a pure microcrystalline powder. A preliminary characterization with an in‐house diffractometer (Figure S1, Supporting Information) confirmed the isolation of the desired form of COF‐300.

**Figure 1:**
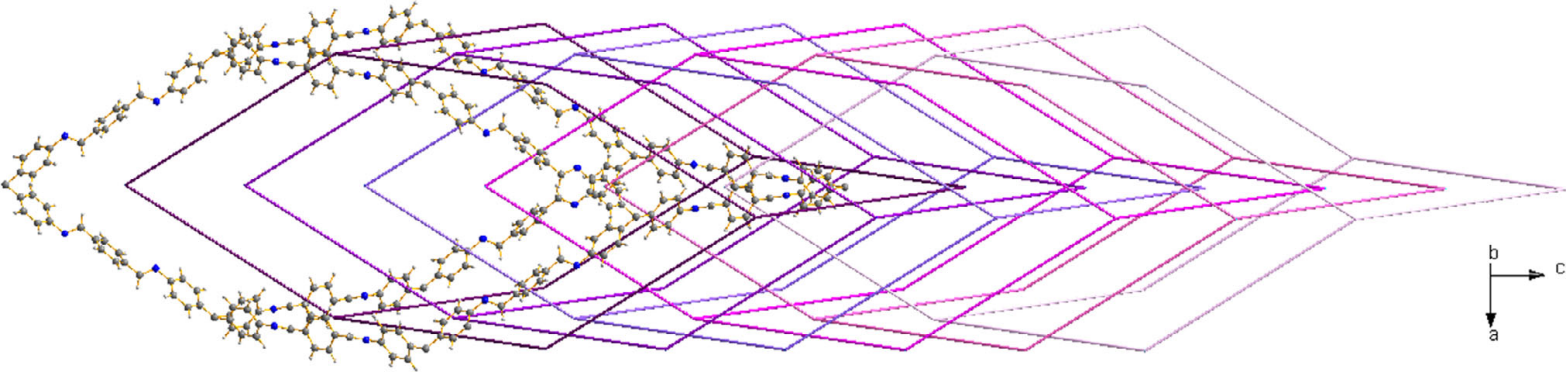
Representation of portion of the 3D framework of the narrow‐pore form of COF‐300 (crystallographic information taken from ref. [28]). The sevenfold interpenetration is schematically depicted from purple to pink. Element color code: *C*, dark grey; *H*, light grey; *N*, blue. The clathrated water molecules have been omitted for clarity.

In its NP^[^ ^26^ ^]^ or LP^[^ ^26^ ^]^ forms, this COF crystallizes in the tetragonal space group *I*4_1_/*a* and possesses a sevenfold interpenetrated 3D network with **dia** topology, featuring 1D linear channels running along the crystallographic *c*‐axis (**Figure** **1**). The channels walls are decorated by the aromatic rings of TAM and BDA (Scheme 1), and the channels width, related to the length of the crystallographic *a*‐axis, depends on the synthesis conditions or external stimuli.

### 2.2 Thermal Behavior of COF‐300

Based on an in‐house temperature‐resolved PXRD (TR‐PXRD) experiment from 298 up to 598 K,^[^ ^29^ ^]^ upon raising the temperature the NP form of COF‐300 undergoes a stepwise increase of the unit cell volume triggered by both thermal expansion and, in the range of 318–338 K, the rupture of the host–guest interactions involving the clathrated water molecules.^[^ ^28^ ^]^

The gas handling system available at ID22^[^ ^31^ ^]^ does not allow to dose vapors of chemicals which are liquid at ambient conditions. Thus, we made use of the custom‐made double‐capillary system described in Section 4.3 (Figure S2, Supporting Information), heating it at specific temperatures to obtain the desired benzene or cyclohexane pressures (Table S1, Supporting Information). The temperatures were calculated through the Antoine equation^[^ ^32^, ^33^ ^]^ (Equation (1)), modeling the relationship existing between the vapor pressure *P* and the temperature *T* of pure substances

(1) $$\log_{10}P=A-\frac{B}{T+C}$$

where *T* and *P* are expressed in Celsius and mmHg, respectively; in the range of 256–353 K: *A* = 6.87987, *B* = 1196.76, and *C* = 219.161 for benzene; *A* = 6.85146, *B* = 1206.47, and *C* = 223.136 for cyclohexane;^[^ ^33^, ^34^ ^]^ in the range of 353–413 K: *A* = 7.2009, *B* = 1415.8, and *C* = 248.028 for benzene; *A* = 7.09926, *B* = 1380.54, and *C* = 246.526 for cyclohexane.^[^ ^33^ ^]^

Given the adopted experimental conditions, during our HR‐NA‐PXRD experiment, COF‐300 was concomitantly exposed to two different external stimuli, namely temperature and adsorbate pressure. With the aim of deconvoluting the influence of the two stimuli on the framework of the COF, HR‐TR‐PXRD patterns (Figure S3a, Supporting Information) were acquired in the temperature range of 256–403 K (Table S2, Supporting Information), in agreement with the thermal range of benzene and cyclohexane dosage. Two NP forms (NP1 and NP2 in the following) and traces of two intermediate pore forms (IP1 and IP2) were identified between 256 and 344 K (Figure S3a, Supporting Information, red traces). The presence of NP1 and NP2, showing slightly different unit cell parameters (Table S2, Supporting Information), and that of IP1 and IP2, with a higher pore aperture than NP1 and NP2 (as evidenced by their *a*‐axis; Table S2, Supporting Information), can be rationalized admitting that different quantities of water molecules entered the pores of the sample. Incidentally, the apparent discrepancy with the preliminary in‐house PXRD characterization (Section 2.1), which did not detect NP2 nor the trace amounts of IP1 and IP2, stems from the higher resolution of the instrumental configuration at work at the beamline ID22. At temperatures higher than 344 K, the four forms coalesced and only NP1 persisted (Figure S3a, Supporting Information, orange traces). As revealed by whole powder pattern refinements with the Le Bail method,^[^ ^35^ ^]^ the crystallographic *a*‐axis and *c*‐axis of NP1 and NP2 increases and decreases, respectively (Table S2 and Figure S4a,b, Supporting Information, for NP1 and NP2, respectively). This occurrence results in an overall expansion of the unit cell volume (up to 10.2% at 389 K and 2.2% at 344 K for NP1 and NP2, respectively). As for IP1 and IP2, due to their very low amount only their first Bragg reflection is visible (highlighted by asterisks in Figure S3b, Supporting Information), so that a quantitative description of their behavior as a function of the temperature is unfeasible.

As we will discuss later, upon dosing benzene and cyclohexane vapors up to four IP forms and two LP forms were detected. Clearly, for these forms it was not possible to deconvolute the influence of temperature from that of the adsorbate pressure, as the former effect could not be directly quantified by means of HR‐TR‐PXRD. We should have admitted that the influence of temperature on these forms was the same as that we observed on NP. Consequently, while treating the HR‐NA‐PXRD data acquired upon dosing the two vapors, as a first approximation we ignored the contribution of temperature on the COF framework.

### 2.3 Behavior of COF‐300 upon Benzene Dosage

**Figure 2:**
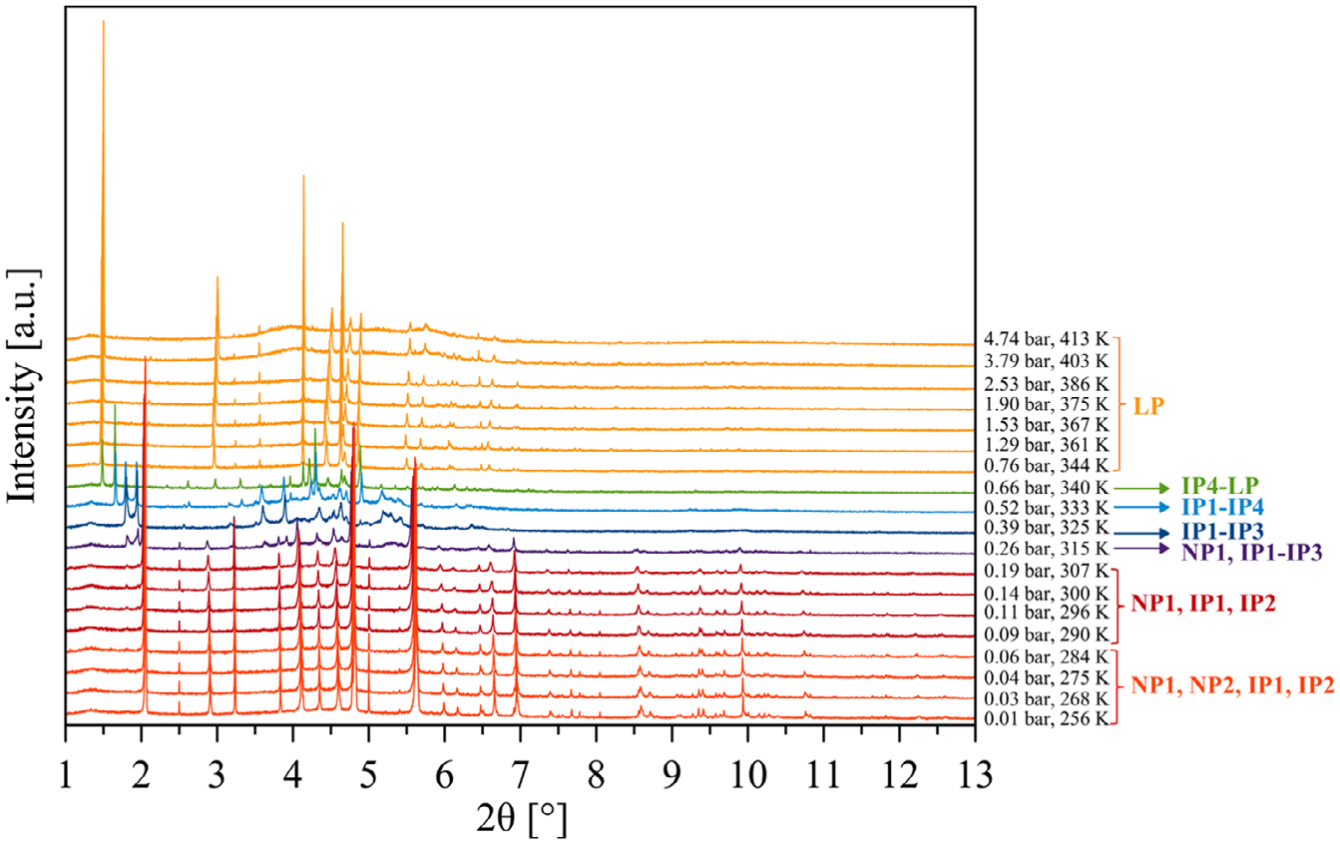
HR‐NA‐PXRD patterns (*λ* = 0.35416 Å) acquired while increasing *P*_BEN_ from 0.01 to 4.74 bar. The legend on the right highlights the forms of COF‐300 present at each pressure/temperature. The appearance of a certain amount of an amorphous phase at 3.79 bar (403 K) is due to the fact that, at this pressure, the double‐capillary system started to wobble. To stabilize it, we covered its basis with wax and flamed it, this action affecting the COF. Indeed, the PXRD pattern acquired at 3.79 bar with the wobbling double‐capillary did not show any contribution from an amorphous phase (not shown), nor amorphization was observed at 403 K during the HR‐TR‐PXRD experiment or during cyclohexane dosage at a similar pressure (see Figure S3a and S7, Supporting Information, respectively).

The structural evolution of COF‐300 while adsorbing liquid benzene was followed by in‐house PXRD:^[^ ^29^ ^]^ after impregnation, a mixture of an IP and an LP form is formed, preliminarily confirming the complexity of the system suggested by volumetric adsorption. To deepen these observations with those emerging from an experiment on a faster timescale and quantitatively dosing benzene and cyclohexane vapors, we recurred to HR‐NA‐PXRD. We dosed benzene vapors on the sample of NP form in our hands in the range of 0.01–4.74 bar (*P*_BEN_). **Figure** **2** collects the acquired data.

A visual comparison of the latter data with those of the HR‐TR‐PXRD experiment (Figure S3a, Supporting Information) confirms that, upon benzene adsorption, COF‐300 shows coherent switchability also when probed with benzene. Indeed, as illustrated in the following, the COF preserves its initial crystallinity and, more importantly, it undergoes a significantly high unit cell parameters modification and a multifaceted framework evolution. Data treatment by means of whole powder pattern refinements with the Le Bail method led to the identification of seven forms (Figure 2) with different tetragonal space groups and unit cell parameters (Table S3, Supporting Information), namely two NP forms (NP1 and NP2), up to four IP forms (IP1–IP4) and one LP form. A rapid evolution of the framework characterizes the range of 0.26–0.66 bar, when the four IP forms are present: in this range, a scan‐by‐scan change of the peak maximum positions and/or the peak intensities is at work, disclosing a system evolution more rapid than the timescale of the experiment and precluding any data treatment but whole powder pattern refinements.^[^ ^36^ ^]^ This complexity is further witnessed by the tetragonal space group (*I*4_1_) of IP4, whose Bragg reflections could not be entirely described by the space group (*I*4_1_/*a*) characterizing the known NP and LP forms (and the other IP forms we observed). Reasonably, the adsorbate distribution in the 1D channels of IP4 does not conform to the higher crystallographic symmetry of the other forms of COF‐300. Remarkably, this is the first time that a form of COF‐300 with crystallographic symmetry lower than the previously known one is disclosed and reported.

**Figure 3:**
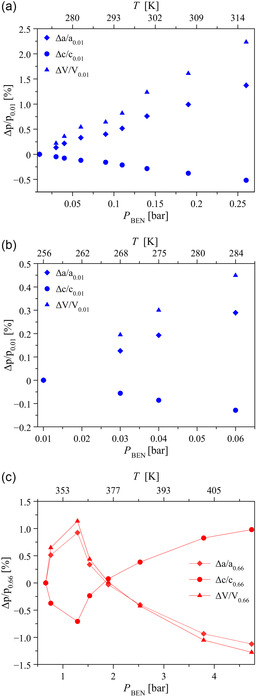
Percentage relative variation of the unit cell parameters of a) NP1, b) NP2, and c) LP as a function of *P*_BEN_ (and temperature) as retrieved with whole powder pattern refinements carried out on the HR‐NA‐PXRD patterns collected in Figure 2. The lines in (c) guide the eye. The reader is addressed to Table S3, Supporting Information, for the values of the unit cell parameters.

**Figure 4:**
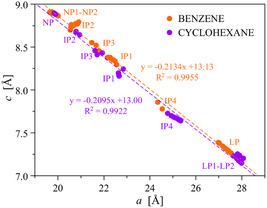
Correlation of the *a*‐axis and *c*‐axis values for the COF‐300 forms identified upon increasing *P*_BEN_ (orange circles) or *P*_CH_ (violet circles), as retrieved with whole powder pattern refinements carried out on the HR‐NA‐PXRD patterns shown in Figure 2 and S7, Supporting Information, respectively. The error bars are not depicted as they would not be visible (even if expressed as 3*σ*). The reader is addressed to Table S3 or S8, Supporting Information, for the values of the two axes while dosing benzene or cyclohexane, respectively.

As evident from Table S3, Supporting Information, upon raising *P*_BEN_ the *a*‐axis and *c*‐axis of all the observed forms increases and decreases, respectively. **Figure** **3** depicts the percentage relative variation of the unit cell parameters of NP1, NP2, and LP as representative examples. Notably, at a first approximation, the changes of the two crystallographic axes are linearly related irrespective of the COF‐300 form (**Figure** **4**). The modification of the *a*‐axis and *c*‐axis overall results in a unit cell volume increase up to 1.29 bar. With reference to NP1 at 0.01 bar (the lower *P*_BEN_ essayed), the unit cell volume expansion amounts to only 2.2% as a maximum for NP1,^[^ ^37^ ^]^ yet it lays in the range of 11–35% in the case of IP1–IP4 and reaches the notable value of 59% for LP (Table S4, Supporting Information), clearly witnessing the adsorbate entrance in IP1–IP4 and LP.

At pressures higher than 1.29 bar LP slightly shrinks, its unit cell volume eventually becoming 1.3% smaller than at the pressure at which it appears (*P*_BEN_ = 0.66 bar). This occurrence could be explained admitting that, after reaching a specific value, the pressure inside the capillary squeezes the COF. As we will comment later, this unit cell shrinking upon pressure increase is associated with benzene desorption. We tend to exclude that this is a case of negative pressure expansion, as proposed by Krause and colleagues for DUT‐49^[^ ^38^ ^]^ and DUT‐50:^[^ ^39^ ^]^ at variance with what shown by their work, apart from the volume decrease, above *P*_BEN_ = 1.29 bar, we did not observe deformations of the framework different from those at work at lower *P*_BEN_ and which could trigger benzene desorption.

**Figure 5:**
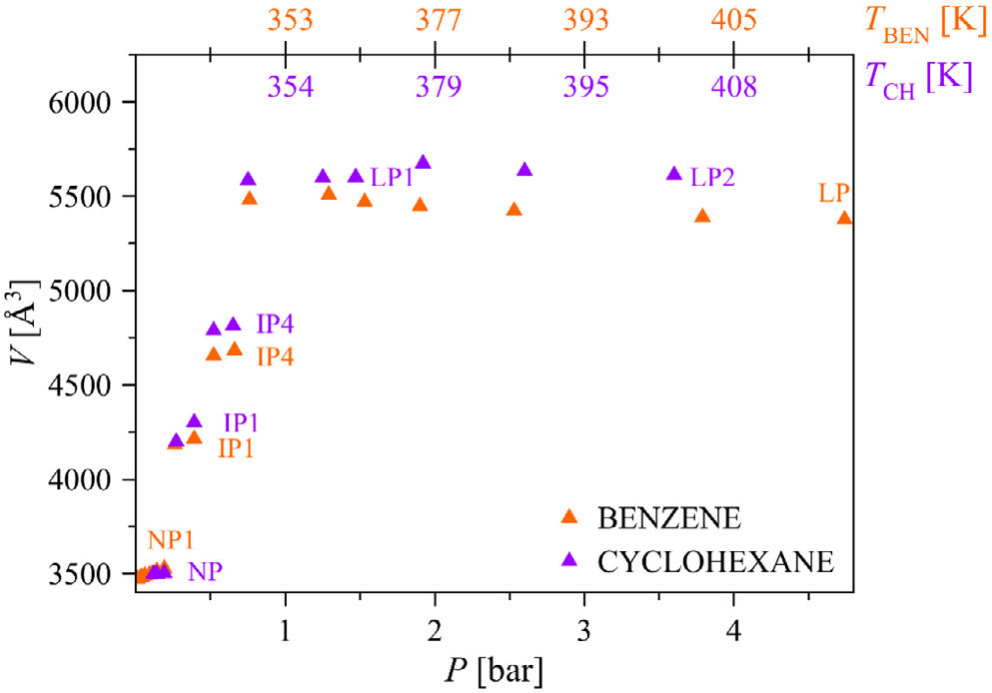
Unit cell volumes of NP1, IP1, IP4, and LP, as representative examples of the forms detected while dosing benzene in the range of 0.01–4.74 bar (orange triangles), and of NP, IP1, IP4, LP1 and LP2, as representative examples of the forms detected while dosing cyclohexane in the range of 0.12–3.60 bar (violet triangles). The error bars are not depicted as they would not be visible (even if expressed as 3*σ*). The reader is addressed to Table S3 or S8, Supporting Information, for the volume values while dosing benzene or cyclohexane, respectively.

Worthy of note, the detection of the IP1–IP4 and LP forms rationalizes the previously observed^[^ ^29^ ^]^ stepwise benzene adsorption isotherm through a stepwise pore aperture from the pristine NP form to the LP one through a number of IP forms, as nicely illustrated in **Figure** **5**.

After identifying the forms of COF‐300 and disclosing the behavior of their unit cell parameters as a function of *P*_BEN_, we located the primary adsorption sites for *P*_BEN_ values in the range of 0.66–4.74 bar, i.e., when only the LP form or a mixture of the IP4 and LP forms are present. As stated earlier, in presence of a mixture of IP forms, the rapid scan‐by‐scan evolution of the peak maximum positions and/or intensities precluded any data treatment but whole powder pattern refinements.

As a first step, we refined the crystal structure of NP1 starting from the known crystallographic information^[^ ^28^ ^]^ and working on the data acquired at *P*_BEN_ = 0.19 bar (the highest pressure at which this form is almost pure). Based on the structural characterization approach described in Section 4.3, we can safely conclude that no adsorbate enters the pores of NP1 which, consequently, only hosts water molecules. The unit cell volume increase characterizing this form upon benzene dosage (2.2% as a maximum) is thus exclusively due to thermal expansion and water release (with concomitant host–guest hydrogen bonds breaking) promoted by temperature rise. An additional confirmation is obtained comparing the change of the unit cell volume of NP1 while exclusively increasing the temperature and while dosing benzene (Figure S5, Supporting Information). Initially increasing at the same rate^[^ ^40^ ^]^ and increasing more rapidly from 296 K on while dosing benzene, at 315 K, the unit cell volume eventually reaches a similar value in the two cases, indicating that temperature is the only external stimulus active on NP1 also while dosing benzene.

**Figure 6:**
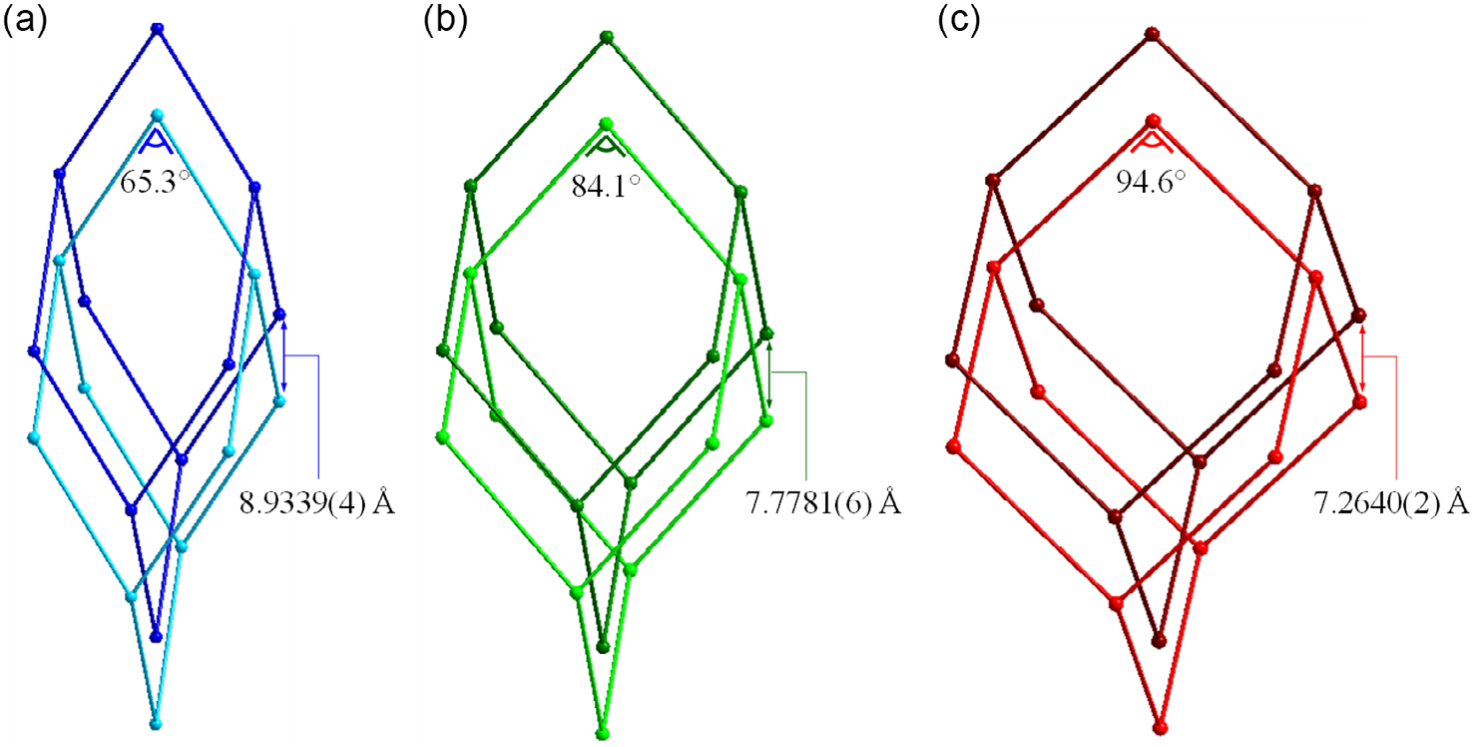
Evolution of the framework of COF‐300 during benzene dosage: a) NP at *P*_BEN_ = 0.19 bar, b) IP4 at *P*_BEN_ = 0.66 bar, and c) LP at *P*_BEN_ = 1.29 bar. Highlighted two of the geometrical parameters discussed in the text, namely the distance between the nearest interpenetrated networks and the angle at the nodes. For all the values of these parameters, the reader is addressed to Table S5, Supporting Information (*c*‐axis), and Table S6, Supporting Information (angle at the nodes).

**Figure 7:**
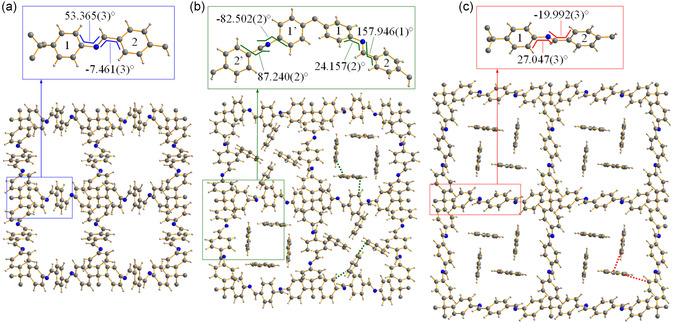
Graphical representation, along the crystallographic *c*‐axis, of portion of the framework of a) NP1 at *P*_BEN_ = 0.19 bar; b) IP4 at *P*_BEN_ = 0.66 bar; and c) LP at *P*_BEN_ = 1.29 bar. The inset highlights the variation of the dihedral angles N1–C8–C9–C10 and C8–N1–C5–C6 triggered by benzene adsorption (for form IP4, whose space groups is *I*4_1_, also the dihedral angles N2–C18–C19–C20 and C18–N2–C15–C16, involving the 1’ and 2’ rings, are reported). Element color code: *C*, dark grey; *H*, light grey; *N*, blue. The clathrated water molecules have been removed for clarity.

**Figure 8:**
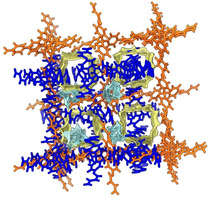
Portion of the framework and of the probe accessible surface^[^ ^44^ ^]^ of NP at *P*_BEN_ = 0.19 bar (blue and light blue, respectively) and of LP at *P*_BEN_ = 1.29 bar (orange and yellow, respectively) at comparison. The reader is addressed also to the short video provided as Supporting Information.

Benzene was successfully located in the pores of IP4 and LP through the Simulated Annealing approach,^[^ ^41^ ^]^ followed by structure refinement with the Rietveld method.^[^ ^42^ ^]^ Table S5–S7, Supporting Information, collect the most salient results of the structure refinements. A gradual enlargement of the pores is at work, as already suggested by the behavior of the unit cell parameters reported above: the empty volume, estimated with the software Mercury,^[^ ^43^ ^]^ increases from ≈26% (in NP at *P*_BEN_ = 0.19 bar) to ≈41% (in IP4 at *P*_BEN_ = 0.66 bar) and ≈50–52% (in LP at *P*_BEN_ = 0.66–4.74 bar) (Table S5, Supporting Information). In addition, the angle at the framework nodes 1) widens from 65.3° to 84.1° and to values in the range of 92.4–94.6° upon passing from NP1 at 0.19 bar to IP4 at 0.66 bar and to LP in the range of 0.66–4.74 bar, respectively (Table S6, Supporting Information, and **Figure** **6**), in line with the type of flexibility expected for a diamondoid network; 2) linearly correlates with the unit cell volume (Figure S6a, Supporting Information). Concomitantly, the distance between the nearest interpenetrated networks (coincident with the crystallographic *c*‐axis) 1) decreases (Table S5, Supporting Information, and Figure 6), confirming that interpenetration provides the flexibility of the framework with an additional degree of freedom; 2) linearly correlates with the angle at the framework nodes (Figure S6a, Supporting Information). Benzene adsorption is also favored by the rotational freedom of the “central” phenyl ring of the asymmetric unit with respect to the “lateral” rings. Indeed, the angle between the root mean square planes of the “lateral” and “central” rings (1 and 2, respectively, in **Figure** **7**) undergoes an “on/off” variation from 31.1° for NP1 at 0.19 bar to 8.0° (and 13.3° considering the other crystallographically independent “lateral” ring) for IP4 at 0.66 bar, and in the range of 8.54–1.48° for LP in the range of 0.66–4.74 bar (Table S6, Supporting Information). This occurrence is reflected, e.g., by value of the torsion angles involving the rings (Figure 7). As a result of these structural changes, the probe accessible surface, estimated with the software MoloVol,^[^ ^44^ ^]^ increases up to ≈120% by passing from NP to LP (**Figure** **8** and Table S6, Supporting Information) and shows a linear relation with the unit cell volume (Figure S6b, Supporting Information).

The previously reported geometrical‐parameter modifications recall those illustrated by Chen and coworkers,^[^ ^28^ ^]^ who nonetheless compared the crystal structure of ex situ obtained samples of COF‐300‐H_2_O, evacuated COF‐300 and COF‐300‐THF. The present investigation goes a step forward, as it proposes evidence of COF‐300 framework dynamics based on an in situ adsorbate‐pressure‐resolved X‐ray diffraction experiment. Indeed, though Chen and coworkers acquired NA‐PXRD patterns while dosing CO_2_, they did not exploit them to extract crystallochemical information. To the best of our knowledge, our work is the first report on the use of NA‐PXRD to dose a gas or a vapor on a COF and extract molecular‐level information therefrom. In the present work, in addition to unveiling the framework structural features cooperating to adsorption, we also located the primary adsorption sites for benzene. Based on the different crystallographic symmetry of IP4 and LP (see before), two and one crystallographically independent benzene molecules, respectively, are invariably present and are located around the 4_1_‐screw axis along which the 1D channels run (Figure 7). In the case of LP, in the range of 0.76–4.74 bar, their center of mass shares very similar *x* and *y* fractional coordinates, while *z* (i.e., their position along the 1D channels) and their overall orientation vary (Table S6, Supporting Information).

Different host–guest and guest–guest interactions were identified (Table S7, Supporting Information). Approximately linear or T‐shaped host–guest interactions of the kind C—H···H—C are present at almost all the essayed pressures (with C···C distance in the range of 2.5–3.3 Å, considering both IP4 and LP; Figure 7b,c). In addition, approximately linear or T‐shaped guest–guest interactions of the kind C—H···H—C are at work (with C···C distance in the range of 2.5–3.0 Å, considering both IP4 and LP; Figure 7b,c). Finally, edge‐to‐face guest–guest C—H···*π* interactions characterize all the *P*_BEN_ values higher than 0.66 bar, while sensible host–guest *π*···H—C interactions are only occasionally detected. Notably, this crystallochemical evidence rules out the hypothesis of the formation of *π*–*π* host–guest interactions favoring the selective adsorption of benzene, which would appear a reasonable speculation based on the number of aromatic rings decorating the pore walls of COF‐300.

**Figure 9:**
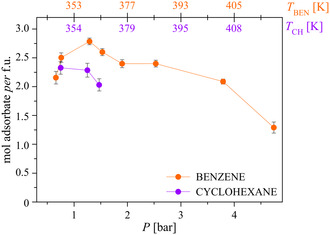
Moles of benzene^[^ ^46^ ^]^ (orange symbols) and cyclohexane (violet symbols) adsorbed per formula unit of COF‐300, as assessed by means of structure refinements performed on the HR‐NA‐PXRD data acquired, respectively, in the *P*_BEN_ and *P*_CH_ range of 0.66–4.74 bar and 0.75–1.47 bar. The error bars represent 3*σ*.

The site occupation factor of the benzene molecules is invariably lower than one and depends on the value of *P*_BEN_. At 0.66 bar, IP4 hosts a total of 2.61(4) mol of adsorbate per mol of IP4.^[^ ^45^ ^]^ With reference to LP, the quantity of adsorbed benzene increases up to 1.29 bar, i.e., while the unit cell volume increases (see before), yielding 2.78(2) mol of benzene per formula unit. Then, when the unit cell volume decreases, also the amount of adsorbate diminishes (Table S6, Supporting Information, and **Figure** **9**).^[^ ^46^ ^]^ To obtain a satisfactory agreement between the experimental and calculated patterns, at any pressure it was necessary to introduce into the pores a number of oxygen atoms (modeling water molecules), whose position was determined by means of the Simulated Annealing approach and whose site occupation factor was refined. At a first approximation, the quantity of clathrated water is inversely correlated with the amount of adsorbed benzene (Table S6, Supporting Information).

### 2.4 Behavior of COF‐300 upon Cyclohexane Dosage

The structural evolution of COF‐300 while adsorbing liquid cyclohexane was followed by in‐house PXRD.^[^ ^29^ ^]^ Immediately after impregnation, a mixture of one NP, two IP, and one LP forms was observed. As a key step forward, HR‐NA‐PXRD patterns were acquired dosing cyclohexane vapors in the range of 0.12–3.60 bar (*P*_CH_) (Figure S7, Supporting Information).^[^ ^47^ ^]^ The material maintains the pristine degree of crystallinity up to the end of the experiment, confirming once more its robustness. Through whole powder pattern refinements with the Le Bail method, seven forms were identified, namely (Figure S7, Supporting Information): one NP form, four IP forms (IP1‐IP4), and two LP forms (LP1 and LP2). Table S8, Supporting Information, collects the values of the unit cell parameters of these forms at any *P*_CH_, while Figure S8, Supporting Information depicts, as representative examples, the percentage relative variation of the unit cell parameters of NP, LP1, and LP2. NP expands by only 0.5% (Table S9, Supporting Information): we can thus exclude also in this case that the adsorbate enters the pores of this form (also considering that cyclohexane has a slightly higher kinetic diameter, *Ø*, than benzene: *Ø*_CH_ = 6.00 Å, *Ø*_BEN_ = 5.85 Å^[^ ^48^ ^]^). Again, the framework undergoes a rapid, scan‐by‐scan evolution in presence of IP1‐IP4, particularly for *P*_CH_ values in the range of 0.27–0.65 bar (matching the *P*_BEN_ range of 0.26–0.66 bar, in which the same phenomenon occurred). The unit cell volume of the IP forms increases upon increasing *P*_CH_ and is 8%–40% larger with respect to that of NP at 0.12 bar (the lowest *P*_CH_ essayed). Notably, at variance with benzene, IP4 persists up to the highest pressure reached (3.60 bar). This occurrence can be taken as a further proof of the higher affinity of COF‐300 for benzene, whose entrance in the COF pores triggers the complete evolution of the system toward the LP form. LP1 and LP2 show unit cell volumes up to 60% and 62% larger than that of NP at 0.12 bar, respectively. Based on their unit cell volume, they are the largest forms of COF‐300 identified during this experiment (and in absolute: their highest unit cell volume amounts to 5601.3(7) and 5673.8(3) Å^3^, respectively [Table S8, Supporting Information], to be compared with the value of 5503.7(5) Å^3^ of COF‐300‐THF^[^ ^28^ ^]^). The larger expansion undergone by COF‐300 upon cyclohexane adsorption can be confidently attributed to its higher steric hindrance, witnessed by its higher kinetic diameter, and does not result in a higher adsorption capacity for this chemical (see below). Both LP forms undergo a contraction starting from 1.92 bar (*P*_CH_ at which LP2 appears) and up to the highest pressure essayed. Worthy of note, also in this case the values of the crystallographic *a*‐axis and *c*‐axis are linearly related irrespective of the COF‐300 form and show the same slope as with benzene (Figure 4). Again, the detection of the IP1–IP4 and LP1–LP2 forms suggests that an expansion from the pristine NP form to the LP ones through a number of IP forms (Figure 5) is at the basis of the observed^[^ ^29^ ^]^ stepwise adsorption isotherm.

After identifying the forms and disclosing the behavior of their unit cell parameters as a function of *P*_CH_, we located the primary adsorption sites working on the three HR‐NA‐PXRD data in the range of 0.75–1.47 bar, when only the IP4 and LP1 forms are copresent. Also in this case, the unit cell volume enlargement is paralleled by an increase of the angle at the framework node (Table S11, Supporting Information). As far as IP4 and LP1 are concerned, the two geometrical parameters are linearly related (Figure S6a, Supporting Information). Cyclohexane was located in the pores of IP4 and LP1 through the Simulated Annealing approach, followed by structure refinement with the Rietveld method. Table S10–S12, Supporting Information, collect the most salient results of the structure refinements. Two and one crystallographically independent cyclohexane molecules, with site occupation factor lower than one and depending on *P*_CH_, are invariably present in IP4 and LP, respectively, and are located around the 4_1_‐screw axis. At variance with benzene, in IP4 the cyclohexane molecules occupy, at the three selected *P*_CH_ values, different primary adsorption sites (as witnessed by the fractional coordinates of their center of mass; see Table S11 and Figure S9, Supporting Information). This higher “mobility” of the guest within the 1D channels, where the primary adsorption site changes from one pressure value to the following one, may concur to the lower adsorption capacity of COF‐300 for cyclohexane. In line with the previous volumetric adsorption studies, the quantity of cyclohexane adsorbed in the range of 0.75–1.47 bar is lower than the quantity of benzene adsorbed at similar pressure values (Table S6 and S11, Supporting Information; Figure 9), notwithstanding the increase in mass percentage of the LP form over IP4 and the fact that the unit cell volume of both forms increases in this pressure range. Host–guest interactions of the kind C—H···H—C are present at all the pressures essayed (with C···C distance of 2.4–3.0 Å, considering both IP4 and LP1; Table S12, Supporting Information). At variance, host–guest interactions of the kind *π*···H—C (1.6–3.1 Å; Table S12, Supporting Information) and guest–guest interactions of the kind C—H···H—C (2.6–3.2 Å; Table S12, Supporting Information) are only occasionally present.

## 3 Conclusions

In situ HR‐PXRD experiments dosing benzene and cyclohexane vapors on the NP form of COF‐300 enabled us to shed light on the dynamics of this soft COF in response to the two chemicals. The material shows coherent switchability, maintaining its crystallinity also above *P*_BEN_ and *P*_CH_ of 3.6 bar, while progressively passing from the pristine NP form to a number of IP and LP forms. Of even higher relevance, we pinpointed key but otherwise inaccessible details, namely progressive and “on/off” structural‐feature changes cooperating in favor of adsorption, primary adsorption sites, and host–guest and guest–guest interactions, which enabled us to successfully complement the previously observed adsorption selectivity of this material toward benzene with key crystallochemical evidence. While, on the short range, our work provides relevant information for the present case of study, on the medium‐long range, it opens the way to new‐generation flexible adsorbents for the applicative context under investigation, and to the use of PXRD in situ dosing gases or vapors to disclose the structural evolution of a COF, an approach here fruitfully adopted for the first time.

## 4 Experimental Section

4.1

4.1.1

##### Materials and Methods

All the reagents and solvents employed for this investigation were purchased from commercial vendors and used without further purification.

##### Synthesis of COF‐300

The synthesis of the NP form of COF‐300 was carried out following the path we recently optimized to isolate it as microcrystalline powders.^[^ ^29^ ^]^ PXRD data to assess the purity and crystallinity degree of the isolated sample were collected at room temperature on a Bruker D8 Advance *θ*–*θ* diffractometer equipped with a sealed X‐ray source (Cu K_α_, *λ* = 1.5418 Å), a Bruker AXS Lynxeye linear position sensitive detector, and the following components: primary‐beam and secondary‐beam Soller slits (2.5°); fixed divergence slit (1 mm); anti‐scatter slit (8 mm); filter of nickel in the diffracted beam. A whole powder pattern refinement with the Le Bail method^[^ ^35^ ^]^ was performed on these data with the software TOPAS‐R v. 3.0^[^ ^49^ ^]^ to confirm the formation of the NP form (Figure S1, Supporting Information).

##### HR‐NA‐PXRD Measurements and Data Treatment

HR‐NA‐PXRD measurements were performed at the ID22 beamline of the ESRF (Grenoble, France; experiment CH‐6220).^[^ ^50^ ^]^ All the data were acquired at 35 keV (*λ* = 0.35416 Å, calibrated at ambient conditions with the National Institute of Standards and Technology standard Si Standard Reference Material 640c), with a beam size of 1.0 × 1.0 mm^2^ defined by water‐cooled slits and made monochromatic with a cryogenically cooled Si 111 channel‐cut crystal. A bank of nine scintillation detectors, each preceded by a Si 111 analyzer crystal, was scanned vertically to measure the diffracted intensity. To increase the signal‐to‐noise ratio, for each temperature/pressure, six scans were acquired, binned, and summed. The subsequent data treatment unveiled that, in some cases, the positions and/or integrated intensities of the Bragg reflections varied from one scan to the other, so that a single scan or a sum of fewer scans than six were used (this occurrence is at the origin of the different values of the figures of merit associated to the data treatment; Table S3 and S8, Supporting Information). To dose benzene and cyclohexane, a custom‐made double‐capillary system containing COF‐300 and the adsorbate in the liquid phase (see the following section and Figure S2, Supporting Information, for further details) was heated at specific temperatures with an Oxford Cryosystems Cryostream 700 plus blower to reach the targeted vapor pressures. The temperatures needed to obtain the latter (in the range of 0.01–4.74 and 0.12–3.60 bar for benzene and cyclohexane, respectively; Table S1, Supporting Information) were calculated using the Antoine equation (Equation (1)).^[^ ^32^, ^33^ ^]^

Aiming to deconvoluting the influence of temperature on the framework softness from that of pressure, a 1 mm diameter borosilicate capillary was filled with the NP form of COF‐300, sealed, mounted on a goniometer head, placed in the diffractometer cradle at ID22, and heated, with the device quoted earlier, at temperatures (Table S2, Supporting Information) similar to those calculated with the Antoine equation for the two adsorbates. HR‐TR‐PXRD data (Figure S3a, Supporting Information) were collected at each temperature in isothermal conditions. To minimize sample radiation damage, the capillary was periodically translated along the goniometric axis during the experiment. For the data collections involving benzene and cyclohexane, a 1 mm diameter Kapton capillary, sealed at one end with a glue solidifying under UV electromagnetic radiation, was filled with the NP form of COF‐300. Some glass wool was then posed on top of the powder to keep it in place. A 0.5 mm diameter Kapton capillary was sealed at one end with the previously quoted glue. Then, it was filled with benzene or cyclohexane. This smaller capillary was introduced in the larger one with the sealed end toward the COF‐300 sample (Figure S2, Supporting Information). Finally, the whole system was sealed with the previously quoted glue and maintained in a refrigerator till the beginning of the experiment, to avoid adsorbate evaporation prior to the HR‐NA‐PXRD experiment. To minimize sample radiation damage, the capillaries were periodically translated along the goniometric axis during the experiment. All the data treatments were carried out with the software TOPAS‐R v. 3.0. The HR‐TR‐PXRD data were treated via whole powder pattern refinements with the Le Bail method adopting, as a starting point, the known space group (*I*4_1_/*a*) and unit cell parameters of the NP form^[^ ^26^ ^]^ (the form massively detected during this experiment; see Section 2) and the IP forms.^[^ ^29^ ^]^ As a representative example, Figure S3b, Supporting Information, proposes the graphical result of the final stage of the whole powder pattern refinement carried out on the data acquired at 284 K, while Figure S4, Supporting Information, depicts the percentage relative variation of the unit cell parameters of NP1 and NP2 as a function of the temperature. Details of all the whole powder pattern refinements are reported in Table S2, Supporting Information. The background was described using a Chebyshev polynomial function. The instrumental contribution to the peak shape was modeled using the Fundamental Parameters approach.^[^ ^51^ ^]^ The sample contribution to the peak profile was described using either the Stephen's approach for the tetragonal crystallographic system,^[^ ^52^ ^]^ or a convolution of Lorentzian and Gaussian functions (further convoluted to spherical harmonics when necessary).

During vapor dosage, a preliminary assessment of the COF‐300 form(s) present at each adsorbate pressure and of the values of the unit cell parameters were obtained via whole powder pattern refinements with the Le Bail method. As a starting point, the space group (*I*4_1_/*a* or *I*4_1_) and unit cell parameters of the NP,^[^ ^26^ ^]^ IP,^[^ ^29^ ^]^ and LP^[^ ^26^ ^]^ forms of COF‐300 were used. The space group *I*4_1_ was employed for the form labeled IP4, identified during both benzene and cyclohexane dosage, which shows a lower crystallographic symmetry with respect to the other forms. The background as well as the instrumental and sample contributions to the peak profile were modeled as described earlier. As a representative example, Figure S10, Supporting Information, proposes the graphical result of the final stage of the whole powder pattern refinement for the data acquired at *P*_BEN_ = 0.09 bar and *P*_CH_ = 0.14 bar, respectively. Details of all the whole powder pattern refinements are reported in Table S3 and S8, Supporting Information, for benzene and cyclohexane, respectively.

To prove that the NP form does not adsorb benzene or cyclohexane, its crystal structure was refined working on the data acquired at *P*_BEN_ = 0.19 bar (307 K), starting from the known crystallographic features.^[^ ^28^ ^]^ The framework asymmetric unit was modeled as a rigid body, assigning idealized bond distances and angles.^[^ ^53^ ^]^ Its center of mass was located on the appropriate special position (Wyckoff letter *a*) and oriented to reproduce by symmetry the whole 3D network. The phenyl rings were allowed to rotate along the exocyclic C—C bonds. To correctly describe the peak intensities, it was necessary to introduce a number of oxygen atoms modeling water molecules, which were located in the 1D channels of the framework working in the real space with the Simulated Annealing approach.^[^ ^41^ ^]^ After localizing the water molecules, the instrumental and structural parameters maintained fixed during the previous stage were collectively refined with the so‐called Rietveld method.^[^ ^42^ ^]^ A unique, refined isotropic thermal parameter was assigned to all the atoms. The background was described using a Chebyshev polynomial function. The instrumental contribution to the peak shape was modeled using the Fundamental Parameters approach. The sample contribution to the peak profile was described using the Stephen's approach for the tetragonal crystallographic system. Figure S11, Supporting Information, proposes the graphical result of the final stage of the Rietveld refinement; salient crystallochemical information is reported in the caption to the figure. Attempts to localize benzene molecules instead of water molecules to model the electron density within the pores resulted in unsensible positions, despite the introduction of proper restraints aiming to limit the minimum distances between host and guest atoms at sensible values.

To locate benzene and cyclohexane within the COF pores, only the HR‐NA‐PXRD data for which just one or two forms (IP4 and LP) of COF‐300 were previously identified were treated. As a first step, the bare framework was built up, starting from the crystal structure already published for the LP form,^[^ ^28^ ^]^ and working *ab initio* for the IP4 form, crystallizing in the space group *I*4_1_. Both the framework asymmetric unit and the benzene and cyclohexane molecules were modeled as rigid bodies, assigning idealized bond distances and angles.^[^ ^53^, ^54^ ^]^ The primary adsorption sites of the two vapors were located by means of the Simulated Annealing approach. To obtain a satisfactory accordance between the experimental and calculated patterns, a number of clathrated water molecules were then located in the framework pores with the Simulated Annealing approach, describing them as bare oxygen atoms for simplicity. Finally, all the refinable structural and instrumental parameters were collectively refined with the Rietveld method. A unique, refined isotropic thermal parameter was assigned to all the atoms. The background was described using a Chebyshev polynomial function. The instrumental contribution to the peak shape was modeled using the Fundamental Parameters approach. The sample contribution to the peak profile was described using either the Stephen's approach for the tetragonal crystallographic system or a convolution of Lorentzian and Gaussian functions (further convoluted to spherical harmonics when necessary). As an example, Figure S12 and S13, Supporting Information, collect the graphical result of the final stage of the Rietveld refinement for the data acquired at *P*_BEN_ = 3.79 bar and *P*_CH_ = 0.75 bar, respectively. Details of all the refinements are reported in Table S5 and S7, Supporting Information, for benzene, and in Table S10 and S12, Supporting Information, for cyclohexane. As representative examples, CCDC numbers 2 359 801‐2 359 803 are referred to the crystallographic information files for NP at *P*_BEN_ = 0.19 bar, IP4 at *P*_BEN_ = 0.66 bar, and LP at *P*_BEN_ = 1.29 bar.

## Conflict of Interest

The authors declare no conflict of interest.

## Author Contributions

**Anna Mauri**: Investigation (lead); Writing—review and editing (supporting). **Rebecca Vismara**: Conceptualization (equal); Investigation (lead); Supervision (equal); Writing—original draft (supporting); Writing—review and editing (supporting). **Marco Moroni**: Investigation (supporting); Writing—review and editing (supporting). **Esther Roldán‐Molina**: Investigation (supporting). **Jorge A. R. Navarro**: Conceptualization (equal); Funding acquisition (equal); Writing—review and editing (supporting). **Simona Galli**: Conceptualization (equal); Funding acquisition (equal); Supervision (equal); Writing—original draft (lead); Writing—review and editing (lead).

## Supporting information

Supplementary Material

## Data Availability

The data that support the findings of this study are available from the corresponding author upon reasonable request.
