# Supplementary material for: Investigating the Dynamics of a Soft Crystalline Covalent Organic Framework during Benzene and Cyclohexane Adsorption by in situ Powder X‐ray Diffraction
Source: Small Sci. 2024 Oct 12;4(12):2400277. doi: 10.1002/smsc.202400277 (PMC11935115; doi:10.1002/smsc.202400277)
Supplement: Supplementary file 1 — Supplementary Material [file SMSC-4-2400277-s001.zip › smsc202400277-sup-0001-SuppData-S1/smsc202400277-sup-0001-SuppData-S1.pdf]

Supplementary Information for

Investigating the Dynamics of a Soft Crystalline COF During Benzene and Cyclohexane Adsorption by *in situ* Powder X-Ray Diffraction

Anna Mauri, Rebecca Vismara, Marco Moroni, Esther Roldán-Molina,*
*Jorge A. R. Navarro, Simona Galli**

A. Mauri, R. Vismara, M. Moroni, S. Galli
Dipartimento di Scienza e Alta Tecnologia, Università degli Studi dell'Insubria
Via Valleggio 9, 22100 Como, Italy
e-mail: simona.galli@uninsubria.it

R. Vismara, E. Roldán-Molina, J. A. R. Navarro
Departamento de Química Inorgánica, Universidad de Granada
Avenida Fuentenueva S/N, 18071 Granada, Spain
e-mail: rvismara@ugr.es

M. Moroni
Dipartimento di Chimica, Università degli Studi di Pavia
Via Taramelli 12, 27100 Pavia, Italy

S. Galli, M. Moroni
Consorzio Interuniversitario Nazionale per la Scienza e la Tecnologia dei Materiali
Via Giusti 9, 50121 Firenze, Italy

Index

Figure S1……………………………………………………………………………pag. S3

Figure S2……………………………………………………………………………pag. S3

Figure S3……………………………………………………………………………pag. S4

Figure S4……………………………………………………………………………pag. S5

Figure S5……………………………………………………………………………pag. S6

Figure S6……………………………………………………………………………pag. S7

Figure S7……………………………………………………………………………pag. S8

Figure S8……………………………………………………………………………pag. S9

Figure S9……………………………………………………………………………pag. S10

Figure S10…………………………………………………………………….….pag. S11

Figure S11…………………………………………………………………………...pag. S12

Figure S12…………………………………………………………………………...pag. S13

Figure S13…………………………………………………………………………...pag. S14

Table S1…………………………………………………………………….….pag. S15

Table S2……………………………………………………………..……………pag. S16

Table S3…………………………………………………………..………………pag. S17

Table S4……………………………………………………………...……………pag. S18

Table S5……………………………………………………………..……………pag. S19

Table S6……………………………………………………………..……………pag. S20

Table S7……………………………………………………………..……………pag. S21

Table S8……………………………………………………………….…..……pag. S22

Table S9…………………………………………………………………….….pag. S23

Table S10..………………………………………………………………….….pag. S24

Table S11..………………………………………………………………….….pag. S25

Table S12..………………………………………………………………….….pag. S26

References………………………………………………………..……………………pag. S26

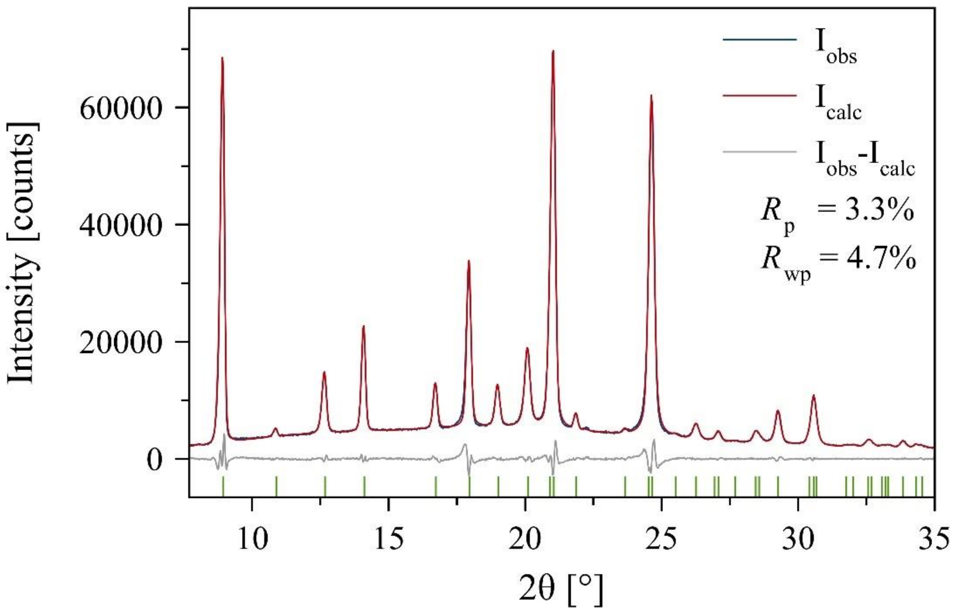

Figure S1. Whole powder pattern refinement, carried out with the Le Bail approach[1] on the PXRD pattern (λ = 1.5418 Å) of the sample of the NP form of COF-300 used for in this work. Observed, calculated, and different patterns in blue, red, and grey, respectively. The green ticks at the bottom indicate the positions of the Bragg reflection maxima.

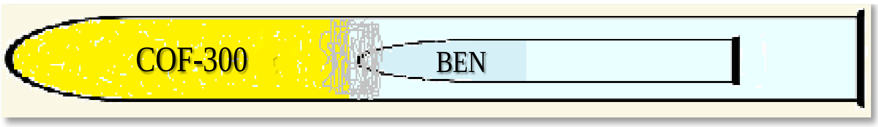

Figure S2. Schematic representation of the custom-made double-capillary system adopted to dose benzene or cyclohexane vapours during the HR-NA-PXRD experiment reported in the main text.

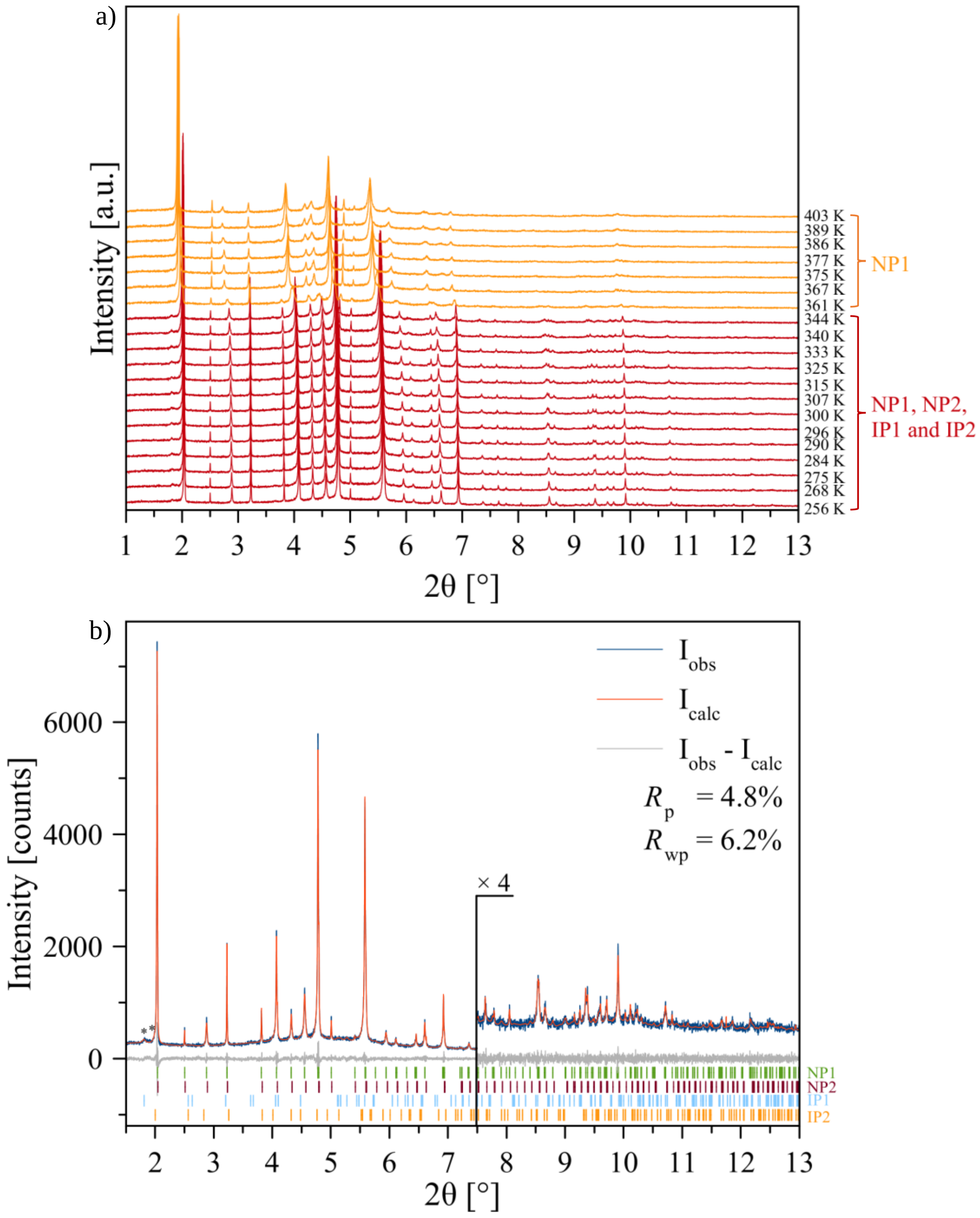

Figure S3. a) HR-TR-PXRD patterns (λ = 0.35416 Å) acquired while increasing the temperature from 256 to 403 K. The legend on the right highlights the forms of COF-300 present at each temperature. b) Graphical result of the final stage of the whole powder pattern refinement carried out with the Le Bail method[1] on the data acquired at 284 K, as representative example. Observed, calculated and difference patterns, blue, red, and grey, respectively. The ticks at the bottom indicate the position of the Bragg reflection maxima for the four forms identified. The asterisks show the first (and only visible) Bragg reflections of forms IP1 and IP2. For the details of all the refinements, the reader is addressed to **Table S2**.

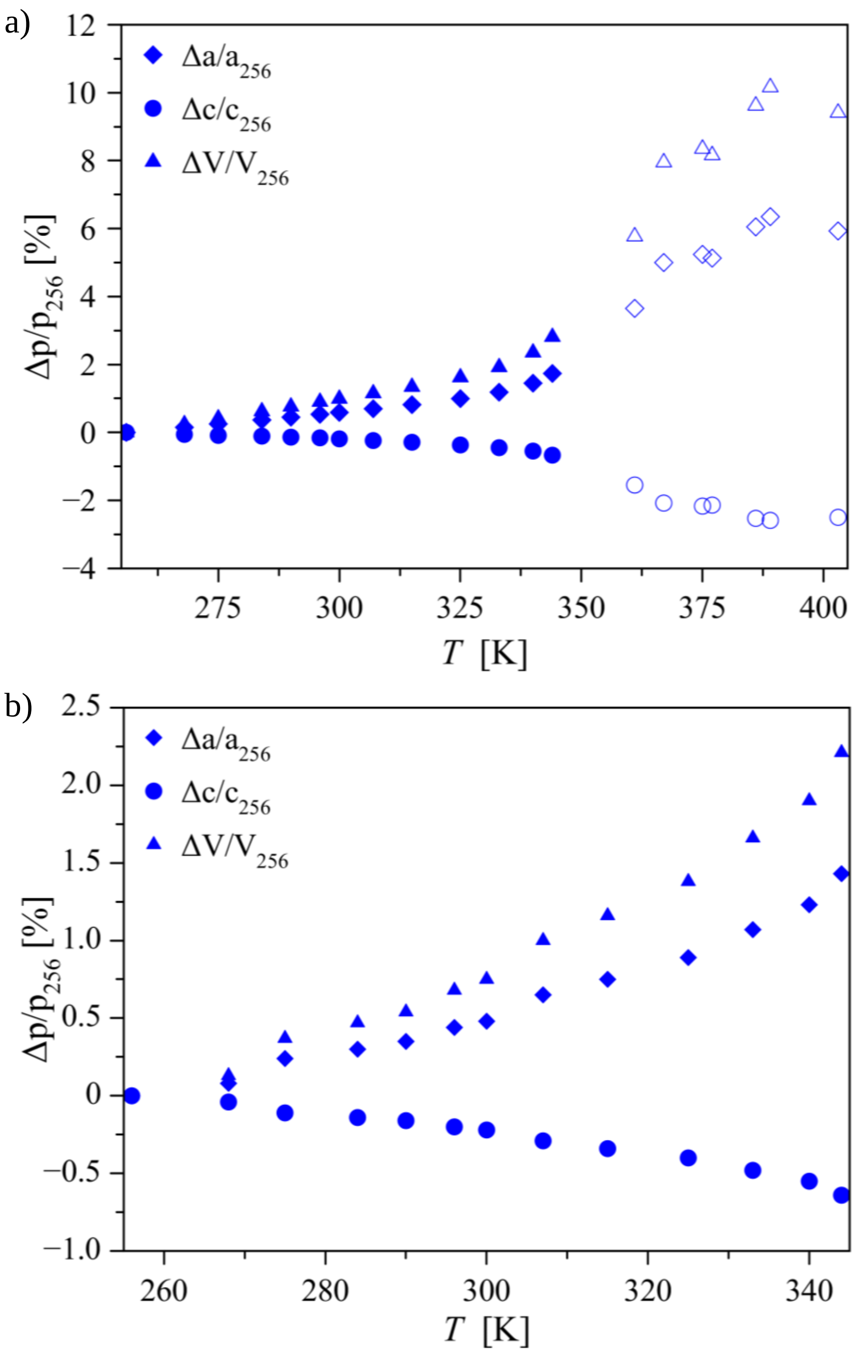

Figure S4. Percentage relative variation of the unit cell parameters of form a) NP1 and b) NP2 as a function of the temperature, as retrieved from the HR-TR-PXRD patterns collected in **Figure S3**a. The empty symbols in a) highlight the temperature values at which NP1 is the only form present. The reader is addressed to **Table S2** for the values of the unit cell parameters.

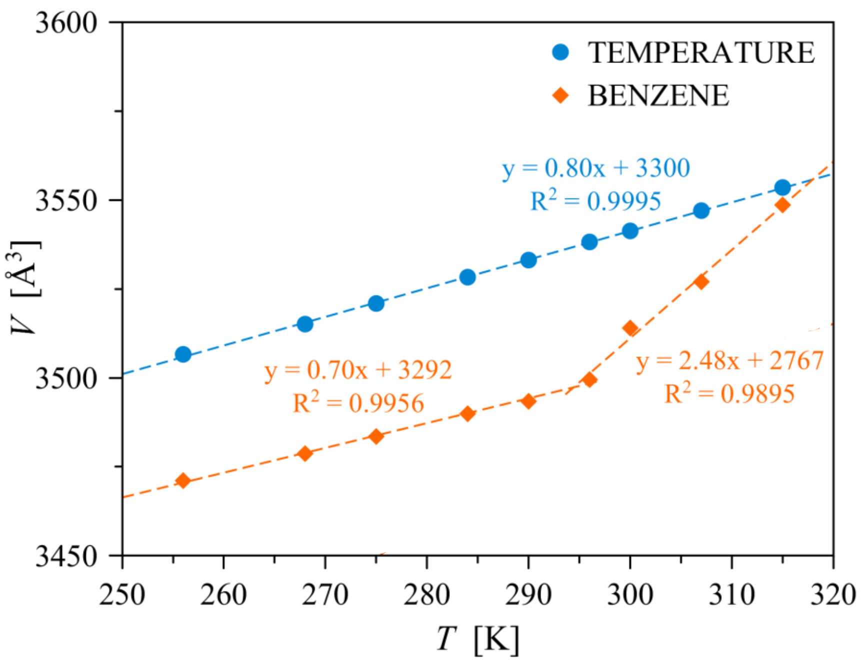

Figure S5. Behaviour of the unit cell volume of NP1 while increasing the temperature and while dosing benzene, as assessed by whole powder pattern refinements with the Le Bail method.[1] The unit cell volume value retrieved at 256 K during benzene dosage is lower than that calculated during the HR-TR-PXRD experiment, probably because the degree of hydration of the sample of COF-300 under investigation increased due to water adsorption from air humidity. The error bars are not reported as they would not be visible (even if expressed as 3σ). For the values of the unit cell volumes, the reader is addressed to **Table S2** and **S3**.

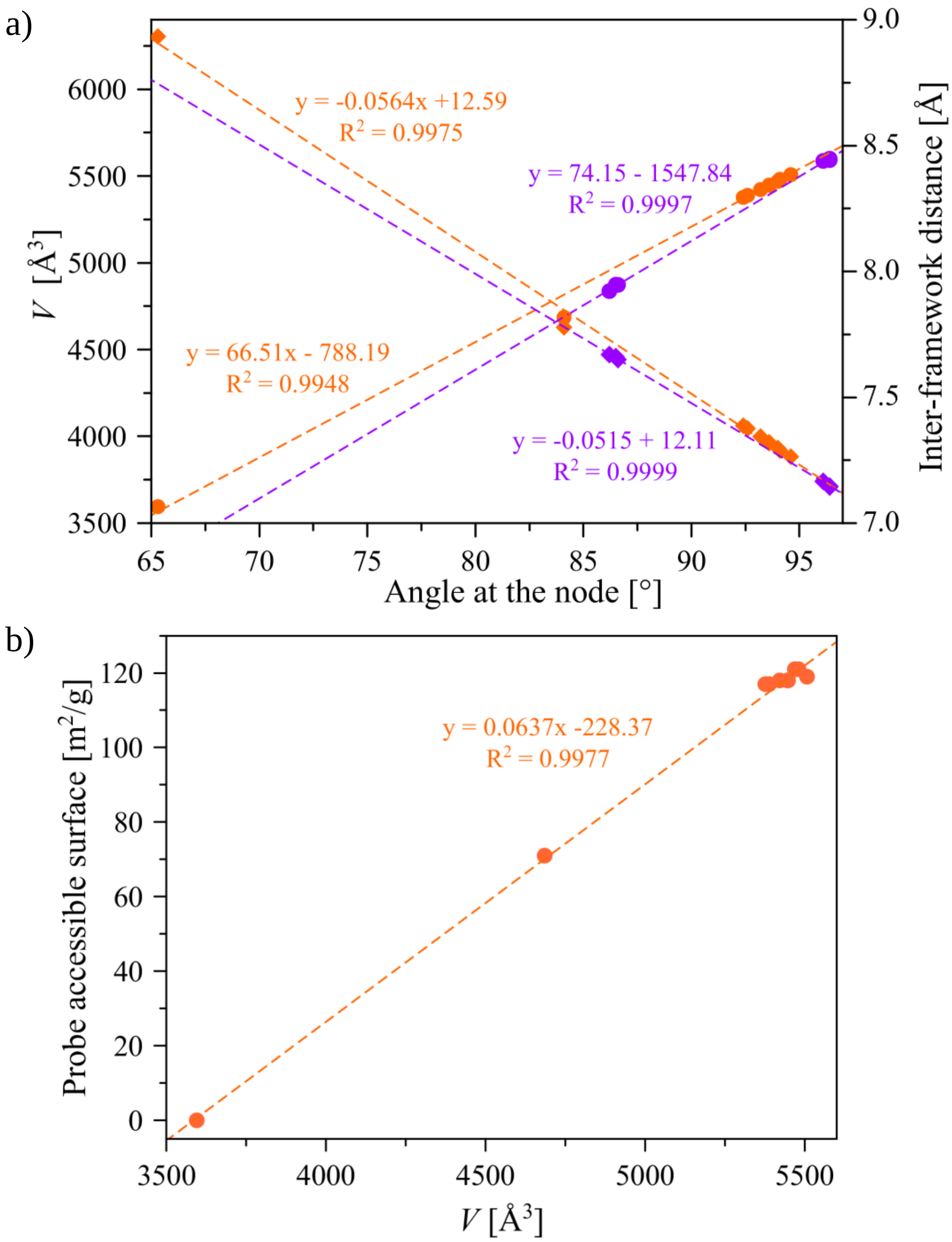

Figure S6. a) Correlation among the amplitude of the angle at the framework node (horizontal axis) and the unit cell volume (circles; left vertical axis) or the inter-framework distance (diamonds; right vertical axis) for the NP, IP4 and LP forms while dosing benzene (orange symbols; **Table S5** and **S6**) and for the IP4 and LP forms while dosing cyclohexane (violet symbols; **Table S10** and **S11**). b) Correlation between the unit cell volume (**Table S5**) and the percentage relative variation of probe accessible surface[2] (**Table S6**) for the NP, IP4 and LP forms while dosing benzene.

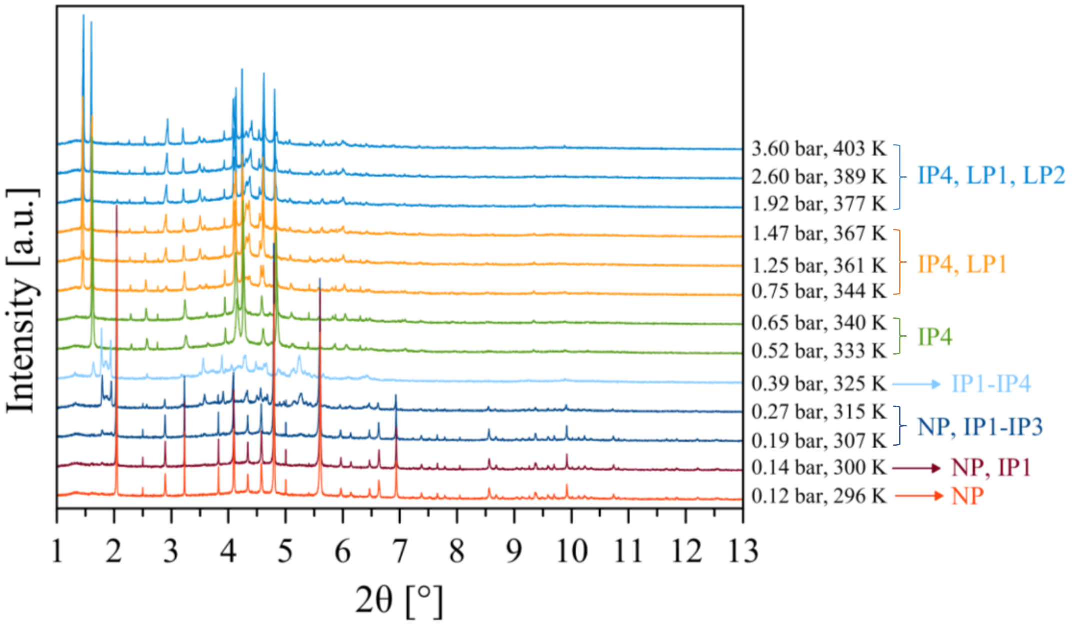

Figure S7. HR-NA-PXRD patterns (λ = 0.35416 Å) acquired while increasing P_{CH} from 0.12 to 3.60 bar. The legend on the right highlights the forms of COF-300 present at each pressure (temperature).

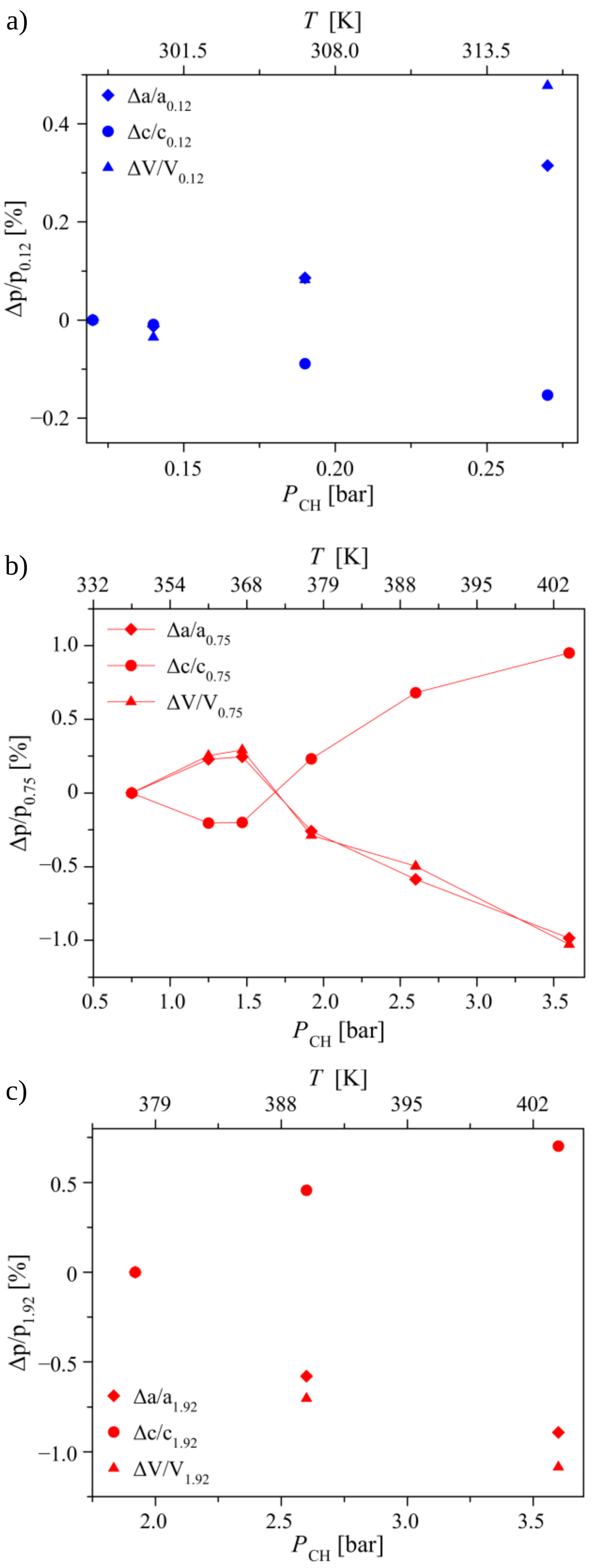

Figure S8. Percentage relative variation of the unit cell parameters of a) NP, b) LP1 and c) LP2 as a function of P_{CH} (and temperature), as retrieved from the HR-NA-PXRD patterns collected in **Figure S7**. The lines in b) guide the eye. The reader is addressed also to **Table S8**.

a)

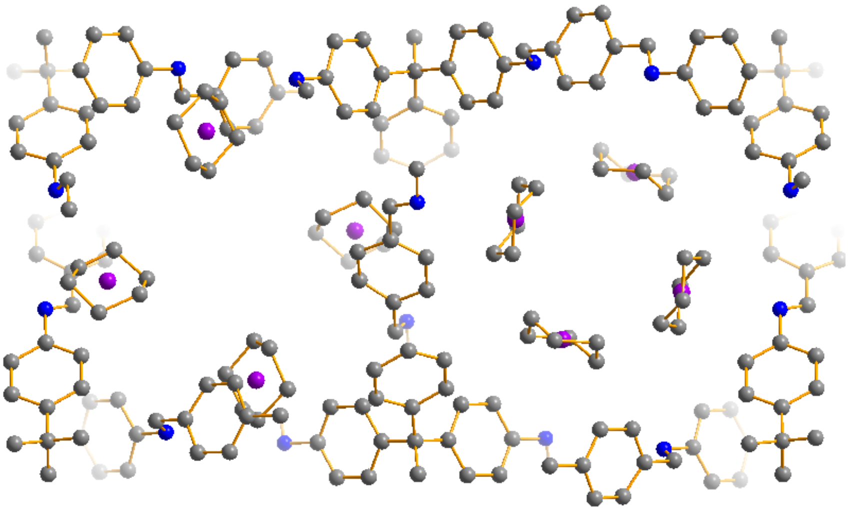

b)

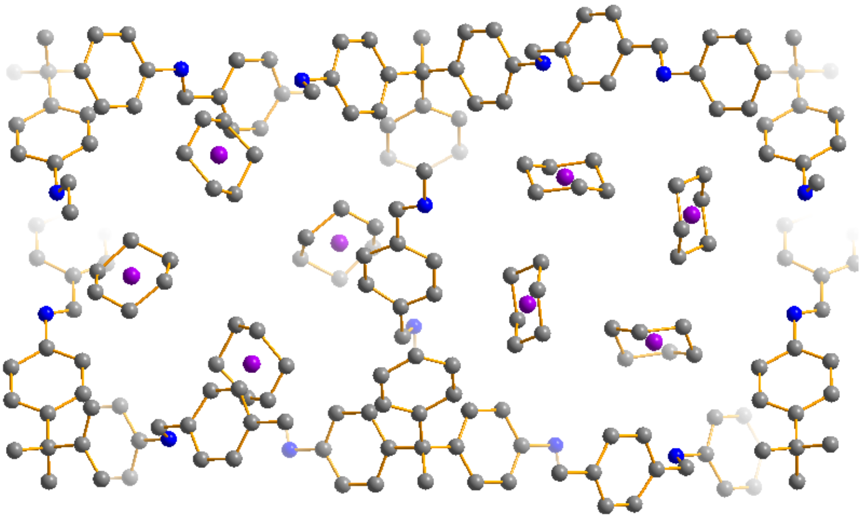

Figure S9. Representation, along the crystallographic *c*-axis, of portion of the framework of form IP4 at P_{CH} of a) 1.25 bar and b) 1.47 bar. Element colour code: C, dark grey; N, blue. Violet dummy atoms show the positions of the centre of mass of the two independent cyclohexane molecules. Clathrated water molecules and hydrogen atoms have been removed for clarity.

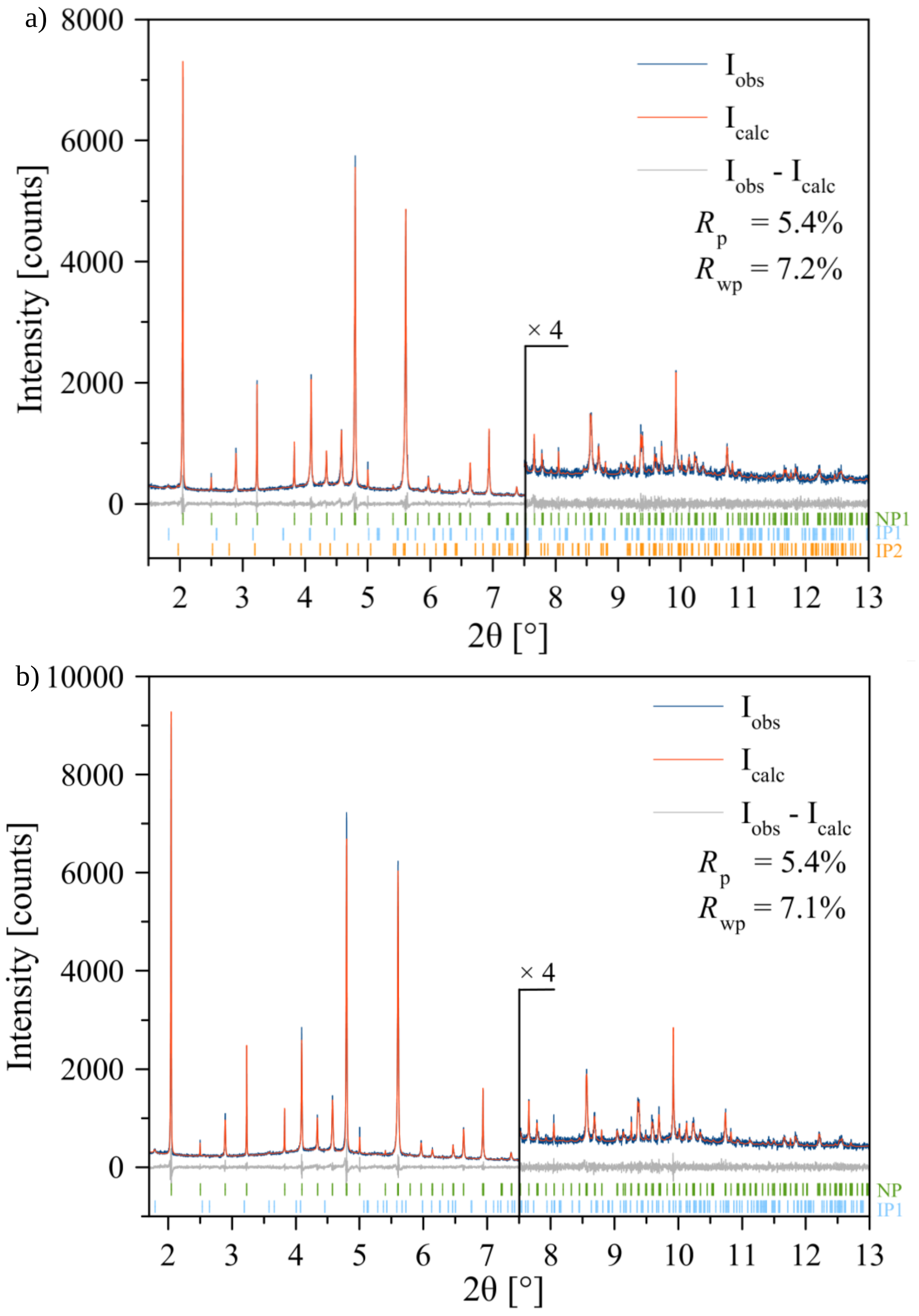

Figure S10. Graphical result of the final stage of the whole powder pattern refinement carried out with the Le Bail method[1] on the HR-NA-PXRD data acquired at a) $P_{\mathrm{BEN}} = 0.09$ bar and b) $P_{\mathrm{CH}} = 0.14$ bar as representative examples. Observed, calculated and difference patterns, blue, red and grey, respectively. The ticks at the bottom indicate the position of the Bragg reflection maxima for the identified forms. The portion above *ca.* 7.5° has been magnified for clarity. For the details of all the refinements, the reader is addressed to **Table S3** and **S8**.

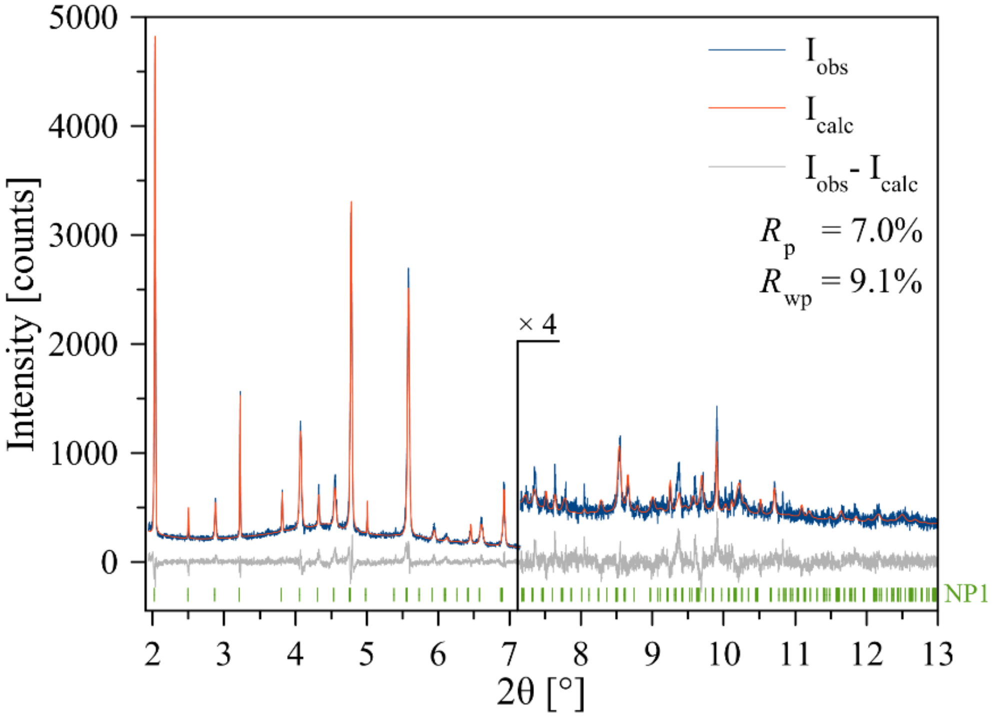

Figure S11. Graphical result of the final stage of the structure refinement for NP1 carried out with the Rietveld method[3] on the HR-NA-PXRD data acquired at P_{BEN} = 0.19 bar. Observed, calculated and difference patterns, blue, red, and grey, respectively. The ticks at the bottom indicate the position of the Bragg reflection maxima. The portion above *ca*. 7.2° has been magnified for clarity.

Salient crystallochemical details: [TAM(BDA)$_2$(H_2O)$_{9.1}$], $C_{41}H_{46.2}N_4O_{9.1}$, FW = 740.71 a.m.u., tetragonal, $I4_1/a$, P_{BEN} = 0.19 bar, T = 307 K, a = 20.060(9) Å, c = 8.934(4) Å, V = 3595(3) Å^3, Z = 4, Z' = 0.25, ρ = 1.37 g cm^{-3}, F(000) = 1572, R_{Bragg} = 3.9%, R_p = 7.0% and R_{wp} = 9.1%, for 5531 data and 54 parameters in the 1.9-13.0° (2θ) range. CCDC number: 2359801.

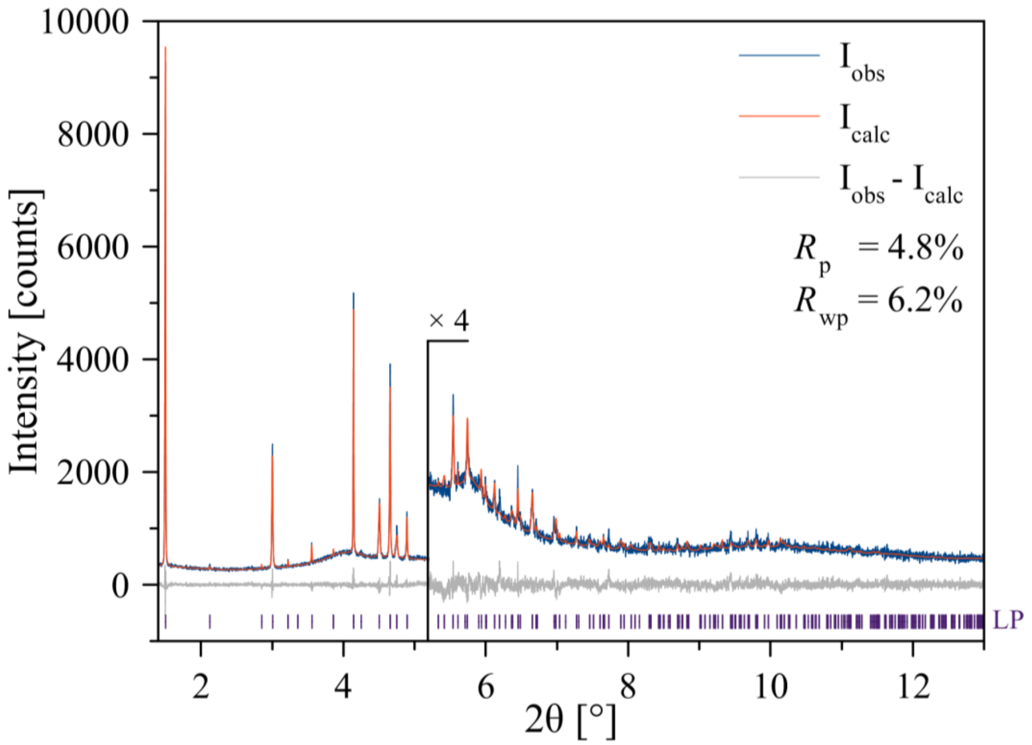

Figure S12. Graphical result of the final stage of the structure refinement for LP1 carried out with the Rietveld method[3] on the HR-NA-PXRD data acquired at P_{BEN} = 3.79 bar. Observed, calculated and difference patterns, blue, red, and grey, respectively. The portion of the pattern above *ca.* 5.2° has been magnified for clarity. The ticks at the bottom indicate the position of the Bragg reflection maxima. For the details of all the refinements, the reader is addressed to **Table S5** and **S6**.

Salient crystallochemical details: $[TAM(BDA)_2(BEN)_{2.1}(H_2O)_{7.3}]$, $C_{53.6}H_{55.2}N_4O_{7.3}$, FW = 872.33 a.m.u., tetragonal, $I4_1/a$, P_{BEN} = 3.79 bar (T = 403 K), a = 27.0292(8) Å, c = 7.3761(2) Å, V = 5388.8(4) Å^3, Z = 4, Z' = 0.25, ρ = 1.08 g cm^{-3}, F(000) = 1852.8, R_{Bragg} = 1.9%, R_p = 4.8% and R_{wp} = 6.2%, for 5801 data and 59 parameters in the 1.4-13.0° (2θ) range. CCDC number: 2359803.

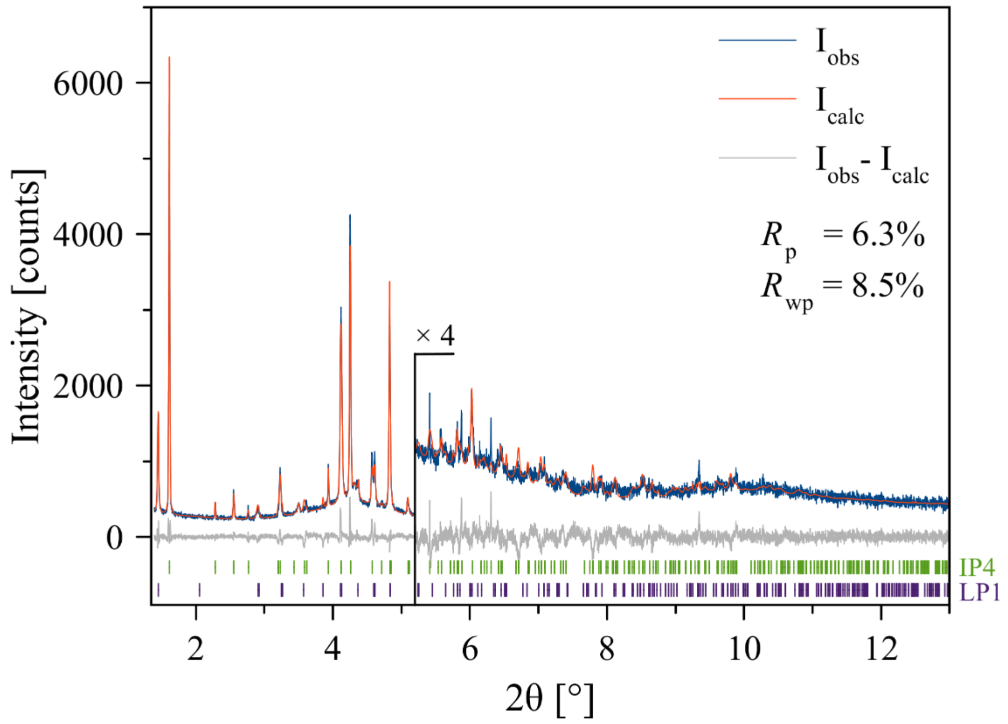

Figure S13. Graphical result of the final stage of the structure refinement for IP4 and LP1 carried out with the Rietveld method[3] on the HR-NA-PXRD data acquired at P_{CH} = 0.75 bar. Observed, calculated and difference patterns, blue, red, and grey, respectively. The portion of the pattern above *ca*. 5.2° has been magnified for clarity. The ticks at the bottom indicate the position of the Bragg reflection maxima. For the details of all the refinements, the reader is addressed to **Table S10** and **S11**.

Salient crystallochemical details form IP4: [TAM(BDA)$_2$(CH)$_{2.0}$(H$_2$O)$_9$], $C_{53}H_{70}N_4O_9$, FW = 907.27 a.m.u., tetragonal, $I4_1$, P_{CH} = 0.75 bar (T = 344 K), a = 25.112(2) Å, c = 7.6682(6) Å, V = 4836.6(8) Å^3, Z = 4, Z' = 0.5, ρ = 1.25 g cm^{-3}, F(000) = 1952, R_{Bragg} = 5.9%, R_p = 6.3% and R_{wp} = 8.5%, for 5086 data and 106 parameters in the 1.4-13.0° (2θ) range.

Salient crystallochemical details form LP1: [TAM(BDA)$_2$(CH)$_{3.0}$(H$_2$O)$_{11}$], $C_{59}H_{86}N_4O_{11}$, FW = 1027.49 a.m.u., tetragonal, $I4_1/a$, P_{CH} = 0.75 bar (T = 344 K), a = 27.922(2) Å, c = 7.1659(2) Å, V = 5587(1) Å^3, Z = 4, Z' = 0.25, ρ = 1.22 g cm^{-3}, F(000) = 2224, R_{Bragg} = 3.0%, R_p = 6.3% and R_{wp} = 8.5%, for 5086 data and 106 parameters in the 1.4-13.0° (2θ) range.

Table S1. Values of temperatures and corresponding values of P_{BEN} and P_{CH}, calculated with the Antoine equation,[4] (**Equation 1** in the main text) at which the HR-NA-PXRD data were acquired.

Benzene	
T [K]	P_{BEN} [bar]
256	0.01
268	0.03
275	0.04
284	0.06
290	0.09
296	0.11
300	0.14
307	0.19
315	0.26
325	0.39
333	0.52
340	0.66
344	0.76
361	1.29
367	1.53
375	1.90
386	2.53
403	3.79
413	4.74

Cyclohexane	
T [K]	P_{CH} [bar]
296	0.12
300	0.14
307	0.19
315	0.27
325	0.39
333	0.52
340	0.65
344	0.75
361	1.25
367	1.47
377	1.92
389	2.60
403	3.60

Table S2. Values of the unit cell parameters, retrieved *via* whole powder pattern refinements with the Le Bail method,[1] for the NP1 and NP2 forms of COF-300 identified raising the temperature in the range 256–403 K.

Form	T [K]	a [Å]	c [Å]	V [Å^3]	R_p, R_{wp} [%]
NP1	256	19.8675(6)	8.8838(2)	3506.6(2)	4.7, 6.1
	268	19.8971(4)	8.8789(2)	3515.1(2)	4.8, 6.2
	275	19.9168(4)	8.8758(2)	3520.9(2)	4.8, 6.2
	284	19.9400(3)	8.8739(2)	3528.3(1)	4.8, 6.2
	290	19.9563(4)	8.8715(2)	3533.1(1)	5.0, 6.4
	296	19.9731(4)	8.8694(2)	3538.2(2)	5.1, 6.6
	300	19.9841(4)	8.8673(2)	3541.3(2)	5.1, 6.6
	307	20.0058(4)	8.8624(2)	3547.0(1)	4.8, 6.2
	315	20.0295(4)	8.8577(2)	3553.5(2)	5.1, 6.5
	325	20.0648(4)	8.8513(2)	3563.5(2)	4.9, 6.2
	333	20.1024(4)	8.8441(2)	3573.9(2)	5.0, 6.6
	340	20.1552(5)	8.8345(2)	3588.9(2)	5.0, 6.5
	344	20.2121(7)	8.8242(3)	3604.9(3)	5.2, 6.7
	361[a)]	20.593(2)	8.746(1)	3708.9(8)	11.3, 14.3
	367[b)]	20.860(1)	8.6986(5)	3785.2(4)	7.2, 9.1
	375	20.9076(9)	8.6913(4)	3799.2(4)	5.6, 7.1
	377	20.887(1)	8.6936(4)	3792.6(4)	5.7, 7.2
	386[c)]	21.069(2)	8.6594(6)	3844.0(6)	8.4, 10.7
	389	21.128(1)	8.6532(4)	3862.8(4)	5.7, 7.3
	403[a)]	21.046(2)	8.6619(8)	3836.7(9)	11.1, 14.4
NP2	256	19.8026(7)	8.8739(2)	3479.8(2)	4.7, 6.1
	268	19.8190(8)	8.8706(2)	3484.3(2)	4.8, 6.2
	275	19.8494(6)	8.8645(1)	3492.6(2)	4.8, 6.2
	284	19.8620(6)	8.8619(1)	3496.0(2)	4.8, 6.2
	290	19.8713(6)	8.8600(1)	3498.6(2)	5.0, 6.4
	296	19.8896(5)	8.8563(1)	3503.5(1)	5.1, 6.6
	300	19.8982(7)	8.8546(1)	3505.9(2)	5.1, 6.6
	307	19.9306(6)	8.8480(1)	3514.7(2)	4.8, 6.2
	315	19.9503(5)	8.8441(1)	3520.1(1)	5.1, 6.5
	325	19.9785(6)	8.8383(1)	3527.8(2)	4.9, 6.2
	333	20.0141(7)	8.8311(2)	3537.5(2)	5.0, 6.6
	340	20.0459(7)	8.8247(2)	3546.1(2)	5.0, 6.5
	344	20.085(1)	8.8168(2)	3556.8(3)	5.2, 6.7

[a)] The refinement was performed on a single scan. [b)] The refinement was performed on a pattern obtained by summing three scans. [c)] The refinement was performed on a pattern obtained by summing two scans.

Table S3. Values of the unit cell parameters, retrieved through whole powder pattern refinements with the Le Bail method,[1] for the seven forms of COF-300 identified while increasing P_{BEN} in the range 0.01–4.74 bar.

Form	P_{BEN} [bar]	a [Å]	c [Å]	V [Å^3]	R_p, R_{wp} [%]
NP1	0.01	19.7371(3)	8.9104(1)	3471.1(1)	4.9, 6.4
	0.03	19.7637(2)	8.9061(1)	3478.7(1)	4.8, 6.3
	0.04	19.7799(8)	8.9036(1)	3483.5(3)	4.9, 6.5
	0.06	19.8023(3)	8.8998(1)	3489.9(1)	5.4, 7.1
	0.09	19.8162(4)	8.8962(2)	3493.4(2)	5.4, 7.2
	0.11	19.8389(8)	8.8914(4)	3499.5(3)	5.6, 7.5
	0.14	19.8872(8)	8.8850(2)	3514.0(3)	5.8, 7.6
	0.19	19.9330(7)	8.8768(2)	3527.0(3)	5.1, 6.7
	0.26	20.008(1)	8.8643(5)	3548.6(5)	5.5, 7.0
NP2	0.01	19.6434(5)	8.9061(1)	3436.5(1)	4.9, 6.4
	0.03	19.668(1)	8.9011(2)	3443.2(3)	4.8, 6.3
	0.04	19.681(1)	8.8985(2)	3446.8(3)	4.9, 6.5
	0.06	19.7002(7)	8.8946(2)	3452.0(2)	5.4, 7.1
IP1	0.01	22.146(9)	8.375(6)	4107(4)	4.9, 6.4
	0.03	22.181(8)	8.380(5)	4123(4)	4.8, 6.3
	0.04	22.208(7)	8.379(4)	4133(3)	4.9, 6.5
	0.06	22.251(5)	8.370(4)	4144(3)	5.4, 7.1
	0.09	22.28(1)	8.377(5)	4158(5)	5.4, 7.2
	0.11	22.30(1)	8.368(6)	4160(6)	5.6, 7.5
	0.14	22.40(1)	8.352(6)	4190(6)	5.8, 7.6
	0.19	22.42(1)	8.348(5)	4195(5)	5.1, 6.7
	0.26	22.386(2)	8.350(2)	4184(1)	5.5, 7.0
	0.39[a)]	22.486(3)	8.336(2)	4215(1)	8.5, 11.0
	0.52[b)]	22.557(3)	8.294(2)	4220(2)	7.0, 9.0
IP2	0.01	20.533(5)	8.698(8)	3667(4)	4.9, 6.4
	0.03	20.547(5)	8.701(9)	3673(4)	4.8, 6.3
	0.04	20.56(2)	8.735(7)	3693(6)	4.9, 6.5
	0.06	20.584(7)	8.760(3)	3712(3)	5.4, 7.1
	0.09	20.623(4)	8.747(2)	3720(2)	5.4, 7.2
	0.11	20.644(2)	8.753(1)	3730(1)	5.6, 7.5
	0.14	20.661(3)	8.748(1)	3734(1)	5.8, 7.6
	0.19	20.704(3)	8.754(3)	3753(1)	5.1, 6.7

	0.26	20.772(3)	8.7724(9)	3785(1)	5.5, 7.0
	0.39 [a]	20.850(3)	8.769(1)	3812(1)	8.5, 11.0
	0.52 [b]	20.904(3)	8.792(4)	3842(2)	7.0, 9.0
IP3	0.26	21.47(2)	8.55(3)	3943(14)	5.5, 7.0
	0.39 [a]	21.663(5)	8.520(2)	3998(2)	8.5, 11.0
	0.52 [b]	21.784(5)	8.456(2)	4013(2)	7.0, 9.0
IP4	0.52 [b]	24.342(4)	7.858(2)	4656(2)	7.0, 9.0
	0.66	24.5367(8)	7.7786(5)	4683.1(4)	6.9, 8.8
LP	0.66	27.284(1)	7.3158(8)	5445.9(8)	6.9; 8.8
	0.76	27.423(1)	7.2885(2)	5481.2(5)	6.9, 9.0
	1.29	27.5359(8)	7.2640(2)	5507.8(3)	6.9, 8.9
	1.53	27.3756(8)	7.2985(2)	5469.7(4)	6.6, 8.5
	1.90	27.2754(8)	7.3213(2)	5446.7(4)	6.7, 8.6
	2.53	27.1739(9)	7.3438(2)	5422.8(4)	6.6, 8.6
	3.79	27.0285(8)	7.3762(2)	5388.6(3)	4.4, 5.6
	4.74	26.977(1)	7.3875(3)	5376.4(5)	4.7, 6.0

[a] The refinement was performed on a pattern obtained by summing two scans. [b] The refinement was performed on a pattern obtained by summing three scans.

Table S4. Forms of COF-300 identified during the HR-NA-PXRD experiment dosing benzene and their relative unit cell volume change. $\Delta V = [(V_{\text{form},P} - V_{\text{form},P\text{min}})/V_{\text{form},P(\text{min})}]\times 100$, with P and Pmin = maximum and minimum P_{BEN}, respectively, at which the different forms were identified. In parenthesis, $\Delta V = [(V_{\text{form},P} - V_{\text{NP1},0.01})/V_{\text{NP1},0.01}]\times 100$ is referred to NP1 at 0.01 bar. For the actual values of the unit cell parameters, the reader is addressed to **Table S3**.

Form	P_{BEN} [bar]	ΔV [%]
NP1	0.01-0.26	2.2
NP2	0.01-0.06	0.4
IP1	0.01-0.52	2.7 (22)
IP2	0.01-0.52	4.8 (11)
IP3	0.26-0.52	1.8 (16)
IP4	0.52-0.66	0.6 (35)
LP	0.66-1.29	1.1 (59)
LP	0.66-4.74	-1.3 (55)

Table S5. Salient results of (or deriving from) the structure refinements performed with the Rietveld method[3] on the HR-NA-PXRD data acquired while dosing benzene in the range 0.66–4.74 bar.

P_{BEN} [bar]	Form	S.G.	a [Å]	c [Å]	V [Å^3]	$\Delta V/V_{0.19}$ [%] a)	V_{empty} [Å^3] b)	V_{empty} [%] c)	R_{Bragg} [%]	R_p, R_{wp} [%]	Data	Param.
0.19	NP	$I4_1/a$	20.060(9)	8.934(4)	3595(3)	0	926.5	25.8	3.9	7.9, 9.1	5531	54
0.66	IP4	$I4_1$	24.543(2)	7.7781(7)	4685.3(8)	30.3	1907.3	40.7	4.2	7.3, 9.6	6001	104
	LP	$I4_1/a$	27.285(3)	7.3177(8)	5448(1)	51.5	2790.0	51.2	1.7			
0.76	LP	$I4_1/a$	27.423(1)	7.2884(3)	5481.0(5)	52.5	2805.1	51.2	4.2	8.5, 11.0	5851	50
1.29	LP	$I4_1/a$	27.5355(9)	7.2640(2)	5507.6(4)	53.2	2881.8	52.3	4.4	7.8, 10.2	5851	49
1.53	LP	$I4_1/a$	27.3750(9)	7.2984(2)	5469.4(4)	52.1	2782.1	50.9	3.2	7.4, 9.9	5851	51
1.90	LP	$I4_1/a$	27.2752(9)	7.3212(2)	5446.5(4)	51.5	2801.6	51.4	4.4	7.3, 9.5	5851	53
2.53	LP	$I4_1/a$	27.172(1)	7.3439(3)	5422.0(4)	50.8	2767.2	51.0	3.0	7.3, 9.6	5851	53
3.79	LP	$I4_1/a$	27.0292(8)	7.3761(2)	5388.8(4)	49.9	2706.6	50.2	1.9	4.8, 6.2	5801	59
4.74	LP	$I4_1/a$	26.979(1)	7.3872(3)	5376.9(5)	49.6	2693.9	50.1	1.5	4.6, 5.9	5801	62

a) $\Delta V/V_{0.19} = (V_P - V_{0.19})/V_{0.19}$. b) Estimated with the software Mercury[5] moving, within the crystal structure after the removal of the guests, a probe of radius 1.2 Å along a grid of pace 0.2 Å. c) V_{empty} [%] = V_{empty}/V [%].

Table S6. Salient results of (or deriving from) the structure refinements performed with the Rietveld method[3] on the HR-NA-PXRD data acquired while dosing benzene in the range 0.66–4.74 bar.

P_{BEN} [bar]	Form	PAS [m^2/g] a)	ΔPAS [%] a)	*x* b)	*y* b)	*z* b)	mol BEN *per* f.u. c)	mol H_2O *per* f.u. c)	ω_1 [°] d)	ω_2 [°] e)
0.19	NP	1218	0	n.a.	n.a.	n.a.	n.a.	9.1	65.3	31.1
0.66	IP4	2088	71	0.6348(7)	0.783(1)	0.576(4)	1.56(3)	10.7(3)	84.1	8.3, 10.3
				0.1403(7)	0.763(1)	0.732(6)	1.05(3)			
	LP	2656	118	0.341(3)	0.386(3)	0.30(1)	1.10(8)	16.0(5)	93.6	6.3
0.76		2690	121	0.8439(7)	0.792(1)	0.888(4)	2.50(3)	9.0(2)	94.1	5.2
1.29		2663	119	0.3384(4)	0.3042(8)	0.825(3)	2.78(2)	6.0(1)	94.6	8.5
1.53		2692	121	0.8141(6)	0.1706(4)	0.713(3)	2.60(2)	6.6(2)	94.0	6.6
1.90	LP	2652	118	0.8424(5)	0.769(1)	0.416(3)	2.40(2)	7.5(2)	93.6	7.8
2.53		2651	118	0.1668(5)	0.698(1)	0.001(3)	2.40(2)	7.0(1)	93.2	3.2
3.79		2642	117	0.6670(4)	0.6857(7)	0.960(2)	2.09(1)	7.3(1)	92.6	1.5
4.74		2642	117	0.3296(9)	0.820(1)	0.831(3)	1.29(3)	10.7(2)	92.4	3.6

a) PAS = Probe accessible surface, estimated with the software MoloVol[2] moving, within the crystal structure after removing the guests, a probe of radius 1.2 Å along a grid of pace 0.2 Å. ΔPAS = Percentage relative variation of the probe accessible surface with respect to the value of NP at 0.19 bar. b) *x*, *y*, *z* = Fractional coordinates of the centre of mass of the independent benzene molecule(s). Applying the symmetry operations of space group $I4_1/a$ on the fractional coordinates, equivalence between the *x* values and among the *y* values of the LP form can be assessed in the range 0.76-4.74 bar. c) f.u. = formula unit. At 0.66 bar of benzene, both IP4 and LP are present; considering the mass percentage of the two forms, as assessed by Rietveld refinement, the total amount of benzene and water adsorbed *per* formula unit of COF-300 at this value of P_{BEN} is 2.16(4) and 12.3(3) mol, respectively. d) ω_1 = angle at the framework nodes (see **Figure 6** of the main text). e) Dihedral angle between the "central" and "lateral" rings of the asymmetric unit (see **Figure 7** of the main text). As IP4 crystallizes in the space group $I4_1$, the asymmetric unit is twice that of NP and LP; two dihedral angles must be considered, referred to the two crystallographically independent "lateral" rings. The first value is referred to the root mean square plane of the C2-C3-C4-C5-C6-C7 ring; the second value is referred to the root mean square plane of the C12-C13-C14-C15-C16-C17 ring.

Table S7: Relevant host-guest and guest-guest interactions detected between benzene molecules and COF-300 framework in the pressure range 0.66-4.74 bar. Interactions higher than 3.3 Å or with a non-sensible orientation are not reported.

P_{BEN} [bar]	Form	Host-guest H-C···C-H [Å]	Host-guest π···H [Å]	Host-guest H···π [Å]	Guest-guest H-C···C-H [Å]	Guest-guest H···π [Å]
0.66	IP4	C5d···C20c 2.6	n.a.	H14···π 1.9	C5d···C3d 2.8	H6d···π 3.0
		C5d···C20c 2.5	π···H4g 3.2	H6c···π 2.7	C6g···C1g 2.6	H6g···π 2.4
	LP	C6b···C6 2.5	π···H1b 2.8	H2···π 3.1	n.a.	n.a.
0.76	LP	C5b···C10 3.3	n.a.	H7···π 3.0	C4b···C2b 2.8	H4b···π 2.1
1.29		n.a.	n.a.	n.a.	C6b···C5b 2.9	H4b···π 2.3
1.53		C4b···C4 3.2	n.a.	H7···π 3.3	C6b···C5b 3.0	H1b···π 2.1
1.90		n.a.	n.a.	n.a.	C1b···C4b 2.5	H4b···π 1.9
2.53		C6b···C11 3.1	n.a.	n.a.	C2b···C3b 3.0	H2b···π 2.0
3.79		C1b···C4 3.3	n.a.	n.a.	C4b···C6b 2.8	H4b···π 2.5
4.74		C2b···C4 3.2	π···H3b 3.3	H7···π 3.2	C5b···C5b 3.0	H5b···π 3.2

Table S8. Values of the unit cell parameters, calculated through whole powder pattern refinements with the Le Bail method,[1] for the seven forms identified while increasing P_{CH} in the range 0.12–3.61 bar.

Form	P_{CH} [bar]	a [Å]	c [Å]	V [Å^3]	R_p; R_{wp} [%]
NP	0.12	19.8353(3)	8.8954(1)	3499.8(1)	5.3, 6.8
	0.14	19.8328(3)	8.8946(2)	3498.6(1)	5.4, 7.1
	0.19	19.8523(3)	8.8875(2)	3502.7(1)	5.7, 7.6
	0.27	19.8979(9)	8.8819(4)	3516.6(3)	5.0, 6.6
IP1	0.14	22.662(3)	8.1617(8)	4192(1)	5.4, 7.1
	0.19	22.650(2)	8.1718(9)	4192(1)	5.7, 7.6
	0.27	22.632(2)	8.198(1)	4199(1)	5.0, 6.6
	0.39a)	22.843(2)	8.2435(6)	4301.4(7)	8.5, 10.9
IP2	0.19	20.783(4)	8.675(2)	3747(2)	5.7, 7.6
	0.27	20.790(1)	8.681(1)	3752.3(7)	5.0, 6.6
	0.39 a)	20.942(2)	8.643(1)	3790.4(9)	8.5, 10.9
IP3	0.19	21.611(9)	8.46(1)	3950(6)	5.7, 7.6
	0.27	21.707(8)	8.410(9)	3963(5)	5.0, 6.6
	0.39 a)	21.931(4)	8.431(5)	4055(3)	8.5, 10.9
IP4	0.39 a)	24.774(3)	7.727(1)	4742(1)	8.5, 10.9
	0.52 b)	24.939(2)	7.6992(4)	4788.6(6)	5.8, 7.5
	0.65 b)	25.031(1)	7.6842(4)	4814.6(6)	6.0, 7.8
	0.75	25.124(1)	7.6742(2)	4844.0(4)	4.4, 5.8
	1.25	25.2302(9)	7.6586(3)	4875.2(4)	5.0, 6.3
	1.47	25.2610(9)	7.6539(3)	4884.1(4)	5.2, 6.6
	1.92	25.265(1)	7.6511(4)	4883.7(5)	5.1, 6.6
	2.60	25.3076(9)	7.6462(3)	4897.2(4)	5.3, 6.8
	3.60	25.3382(8)	7.6403(3)	4905.3(4)	5.1, 6.5
LP1	0.75	27.924(2)	7.1623(6)	5585(1)	4.4; 5.8
	1.25	27.988(2)	7.1477(5)	5599.2(7)	5.0; 6.3
	1.47	27.993(1)	7.1480(4)	5601.3(7)	5.2; 6.6
	1.92	27.8521(9)	7.1789(4)	5569.0(5)	5.1; 6.6
	2.60	27.761(1)	7.2111(4)	5557.3(5)	5.3; 6.8
	3.60	27.649(1)	7.2304(6)	5527.6(6)	5.1; 6.5

	1.92	28.066(1)	7.2031(3)	5673.8(3)	5.1; 6.6
LP2	2.60	27.903(1)	7.2359(3)	5633.9(3)	5.3; 6.8
	3.60	27.8157(7)	7.2537(1)	5612.2(2)	5.1; 6.5

[a)] The refinement was performed on a HR-NA-PXRD pattern obtained summing only two scans. [b)] The refinement was performed on a cumulative HR-NA-PXRD pattern obtained summing only three scans.

Table S9. Forms of COF-300 identified during the HR-NA-PXRD experiment dosing cyclohexane and their relative unit cell volume change. $\Delta V = [(V_{form,P} - V_{form,Pmin})/V_{form,Pmin}]\times100$, with P and P_{min} = maximum and minimum value of P_{CH}, respectively, at which the different forms have been identified. In parenthesis, $\Delta V = [(V_{form,P} - V_{NP1,0.12})/V_{NP1,0.12}]\times100$ is referred to NP at 0.12 bar. For the actual values of the unit cell parameters, the reader is addressed to **Table S8**.

Form	P_{CH} [bar]	ΔV [%]
NP	0.12-0.27	0.5
IP1	0.14-0.39	2.6 (23)
IP2	0.19-0.39	1.2 (8)
IP3	0.19-0.39	2.7 (16)
IP4	0.39-3.60	3.4 (40)
LP1	0.75-1.47	0.3 (60)
LP1	0.75-3.60	-1.0 (58)
LP2	1.92-3.60	-1.1 (60) [a)]

[a)] At 1.92 bar, the unit cell volume of LP2 is 62% larger than that of NP at 0.12 bar.

Table S10. Salient results of the structure refinements performed with the Rietveld method[3] on the HR-NA-PXRD data acquired while dosing cyclohexane in the range 0.75-1.47 bar.

P_{CH} [bar]	Form	Space group	a [Å]	c [Å]	V [Å^3]	R_{Bragg} [%]	R_{wp}, R_p [%]	Data	Parameters
0.75	IP4	$I4_1$	25.112(2)	7.6682(6)	4836.6(8)	5.9	8.5, 6.3	5806	106
	LP	$I4_1/a$	27.922(2)	7.1659(2)	5587(1)	3.0			
1.25	IP4	$I4_1$	25.222(2)	7.6617(6)	4873.9(8)	3.8	9.1, 6.8	5951	105
	LP	$I4_1/a$	27.993(2)	7.1470(5)	5600(1)	3.3			
1.47	IP4	$I4_1$	25.238(1)	7.6500(4)	4872.8(6)	5.8	9.3, 6.8	5951	106
	LP	$I4_1/a$	27.978(2)	7.1435(7)	5591.4(9)	2.7			

Table S11. Salient results of the structure refinements performed with the Rietveld method[3] on the HR-NA-PXRD data acquired while dosing cyclohexane in the range 0.75-1.47 bar.

P_{CH} [bar]	Form	x [a)]	y [a)]	z [a)]	mol CH *per* f.u. [b)]	mol H_2O *per* f.u. [b)]	ω_1 [°] [c)]	ω_2 [°] [e)]
0.75	IP4	0.7882(9)<br>0.230(1)	0.875(1)<br>0.6350(8)	0.236(4)<br>0.473(4)	1.95(6)	9.0(3)	86.2	19.2, 17.9
	LP	0.1595(3)	0.2956(5)	0.037(3)	2.95(3)	11.0(3)	96.1	68.0
1.25	IP4	0.418(1)<br>0.2991(9)	0.2839(7)<br>0.8630(7)	0.350(3)<br>0.855(4)	2.63(6)	10.8(3)	86.5	20.6, 19.2
	LP	0.6596(5)	0.8270(6)	0.715(2)	1.77(3)	12.0(3)	96.4	36.1
1.47	IP4	0.7280(9)<br>0.6905(7)	0.8890(8)<br>0.3597(4)	0.595(3)<br>0.977(2)	2.11(4)	9.6(2)	86.6	20.2, 18.7
	LP	0.184(2)	0.849(2)	0.282(8)	1.98(5)	10.5(3)	96.4	41.0

[a)] x, y, z = Fractional coordinates of the centre of mass of the independent cyclohexane molecule(s). [b)] f.u. = formula unit. At each value of pressure, both IP4 and LP are present; considering the mass percentage of the two forms, as assessed by Rietveld refinement, the total amount of cyclohexane and water adsorbed *per* formula unit of COF-300 are (respectively): 2.33(4) and 9.7(2) mol at 0.75 bar; 2.29(4) and 11.3(2) mol at 1.25 bar; 2.03(4) and 10.1(2) mol at 1.47 bar. [c)] ω_1 = angle at the framework nodes (see **Figure 6** of the main text), to be compared with the value of 65.4° for NP1 at P_{BEN} = 0.19 bar. [d)] Dihedral angle between the "central" and "lateral" rings of the asymmetric unit (see **Figure 7** of the main text), to be compared with the value of 31.1° for NP1 at P_{BEN} = 0.19 bar. As IP4 crystallizes in the space group $I4_1$, the asymmetric unit is twice that of NP and LP. Two dihedral angles must be considered, referred to the two crystallographically independent "lateral" rings. The first value is referred to the root mean square plane of the C2-C3-C4-C5-C6-C7 ring; the second value is referred to the root mean square plane of the C12-C13-C14-C15-C16-C17 ring.

Table S12: relevant host-guest and guest-guest interactions detected between cyclohexane molecules and COF-300 framework in the pressure range 0.75-1.47 bar. Interactions longer than 3.3 Å or with non-sensible orientations are not reported.

P_{CH} [bar]	Form	Host-guest H-C···C-H [Å]	Host-guest π···H [Å]	Guest-guest H-C···C-H [Å]
0.75	IP4	C4a···C3b 2.4	π···H5b 1.6	n.a.
		C21a···C2c 2.7	n.a.	C4c···C6c 2.9
	LP	C10···C3d 2.5	n.a.	C6d···C3d 2.8
1.25	IP4	C10a···C5b 2.5	π···H9b 1.6	n.a.
		C21a···C3c 2.8	n.a.	C6c···C2c 2.8
	LP	C4···C5d 3.0	π···H12d 3.1	C3d···C1d 3.2
1.47	IP4	C10a···C1b 2.4	π···H1b 1.9	n.a.
		C6a···C1c 2.6	n.a.	C4c···C6c 2.6
	LP	C1d···C6 2.7	n.a.	C5d···C3d 2.7

References

[1] A. Le Bail, H. Duroy, J. L. Fourquet, *Mater. Res. Bull.* **1988**, *23*, 447-452.

[2] J. B. Maglic, R. Lavendomme *J. Appl. Crystallogr.* **2022**, *55*, 1033-1044.

[3] R. A. Young, IUCr Monograph N. 5, Oxford University Press, New York, USA **1981**.

[4] C. Antoine, *Comptes Rendus des Séances de l'Académie des Sciences* **1888**, *107*, 681-684.

[5] Mercury v. 2024.1.0, Cambridge Crystallographic Data Centre, Cambridge, United Kingdom.
